# Type II grass carp reovirus utilizes autophagosomes for viroplasm formation and subclinical persistent infection

**DOI:** 10.1128/jvi.00352-25

**Published:** 2025-04-02

**Authors:** Qian Wang, Zichao Peng, Pengfei Chu, Bin Gui, Yongming Li, Lanjie Liao, Zuoyan Zhu, Fei Ke, Yaping Wang, Libo He

**Affiliations:** 1 State Key Laboratory of Breeding Biotechnology and Sustainable Aquaculture, Institute of Hydrobiology, Chinese Academy of Sciences 53021 , Wuhan, China; 2 University of the Chinese Academy of Sciences 74519, Beijing, China; 3 College of Animal Science and Technology, Yangzhou University 614678 , Yangzhou, China; 4 Innovative Academy of Seed Design, Chinese Academy of Sciences 645471 , Beijing, China; University of Michigan Medical School, Ann Arbor, Michigan, USA

**Keywords:** type II grass carp reovirus, viroplasms, nonstructural protein NS79, autophagy, autophagosomes, subclinical persistent infection, nonlytic release, viral spread

## Abstract

Grass carp reovirus (GCRV) is the most virulent pathogen within the genus *Aquareovirus*, belonging to the family *Spinareoviridae*. GCRV is categorized into three genotypes, with type II (GCRV-II) being the predominant strain circulating in China. Reoviruses are known to replicate and assemble in cytoplasmic inclusion bodies termed viroplasms; however, information regarding the formation of GCRV-II viroplasms and their specific roles in virus infection remains largely unknown. In this study, we investigated the formation and characteristics of viroplasms during GCRV-II infection. Immunofluorescence and confocal microscopy indicate that GCRV-II infection induces the formation of viroplasms, with the nonstructural protein NS79 being the key protein responsible for this process. Live-cell imaging and fluorescence recovery after photobleaching assays reveal that GCRV-II viroplasms lack liquid-like properties. Transmission electron microscopy confirms that GCRV-II viroplasms are membranous structures. Notably, we demonstrate that GCRV-II infection induces autophagy and the formation of autophagosomes and that GCRV-II utilizes these autophagosomes for viroplasm formation and virion assembly. Furthermore, we found that GCRV-II uses autophagosomes to evade the host immune system, establishing subclinical persistent infection. GCRV-II also employs autophagosomes for nonlytic release and viral spread. Collectively, these findings highlight distinctive characteristics of GCRV-II viroplasms compared to those of other animal reoviruses, offering valuable insights for the prevention and control of this virus.

**IMPORTANCE:**

Grass carp reovirus (GCRV) is categorized into three genotypes, with GCRV-II being the most prevalent in China. Despite reoviruses being known for their replication and assembly in viroplasms, the specifics of GCRV-II viroplasm formation and its role in infection were unclear. Our study demonstrates that GCRV-II infection triggers the formation of viroplasms, primarily mediated by the nonstructural protein NS79. GCRV-II viroplasms are membranous structures that lack liquid-like properties, which are significantly different from the viroplasms of other reoviruses. Notably, our research unveils that GCRV-II infection induces autophagy and utilizes autophagosomes for viroplasm formation and virion assembly. Furthermore, we also confirm that GCRV-II utilizes autophagosomes for subclinical persistent infection, nonlytic release, and viral spread. Our results indicate that GCRV-II hijacks autophagosomes to form viroplasms and complete its life cycle. The characteristics of GCRV-II are significantly different from those of other animal reoviruses, providing important information for prevention and control of this virus.

## INTRODUCTION

Grass carp (*Ctenopharyngodon idella*) is a significant aquaculture species in China, contributing over 21% to the total freshwater aquaculture yield in the country. As of 2022, grass carp production has soared to an impressive 5.90 million tons, underscoring its considerable commercial importance (1). However, the aquaculture of grass carp has been beset by numerous diseases, notably grass carp hemorrhagic disease attributed to grass carp reovirus (GCRV), a significant threat to the aquaculture of the species.

GCRV is the most virulent pathogen within the genus *Aquareovirus*, belonging to the family *Spinareoviridae* (2, 3). Distinguished by genomic banding patterns and sequence variations, GCRV is categorized into three genotypes, with representative strains including GCRV-873 (type I), GCRV-HZ08 (type II), and GCRV-104 (type III) (4). The genome of all GCRVs consists of 11 segments of double-stranded RNA (dsRNA), and the number of encoded viral proteins ranges from 11 to 13 depending on the genotypes (5, 6). Notably, GCRV-873 (type I) was the first fish virus to be characterized and sequenced in China (7) and thus served as a model in early research on disease-resistant breeding, vaccine development, and virus–host interactions. However, numerous studies have shown that most GCRVs isolated in southern China are type II, such as GCRV-HZ08, GCRV-GD108, and GCReV-109 (4, 8, 9), indicating that type II GCRV is the predominant strain currently circulating in China. Type III GCRV is not widely distributed in China, with only one strain identified (GCRV-104) (10). The three types of GCRV have significantly different nucleotide sequences, viral-encoded protein structures, and pathogenicity in grass carp and cultured cells. For instance, type II GCRV infection causes no cytopathic effect (CPE) in CIK cells but induces more than 80% mortality in yearling fish (11). In contrast, type I and III GCRV infections cause obvious cytopathic effects (CPE) in CIK cells but induce mild hemorrhagic disease and mortality in grass carp (10, 12, 13). Nevertheless, the underlying mechanism for these phenomena remains unclear.

Members of the family *Spinareoviridae* are known to replicate and assemble in cytoplasmic inclusion bodies termed viroplasms, which are membraneless structures containing host and viral components essential for viral morphogenesis (14–16). Within the family *Spinareoviridae*, certain viral proteins, such as µNS of mammalian reovirus (MRV) and avian reovirus (ARV) (14, 17), as well as NS80 of type I GCRV (GCRV-I) (15), are crucial for viroplasm formation and recruitment of other viral proteins into viroplasms for virus replication and assembly. Interestingly, recent studies suggest that the viroplasms of some reoviruses exhibit liquid-like properties and are formed through liquid–liquid phase separation (LLPS) of certain viral proteins (18–20). Moreover, our recent study further revealed that the liquid-like properties of reovirus viroplasms are essential for recruiting viral dsRNA, viral RNA-dependent RNA polymerase (RdRp), and viral proteins to participate in viral genome replication and virion assembly, as well as for sequestering host antiviral factors for immune evasion (21). However, as the predominant strain of GCRV circulating in China, information regarding the formation of type II GCRV (GCRV-II) viroplasm formation and their specific roles in virus infection remains largely unknown. Understanding the underlying mechanisms will provide important insights into the pathogenesis of GCRV-II infection, as well as potential targets for its prevention and control.

In this study, we investigated the formation and characteristics of viroplasms during type II GCRV infection. We found that GCRV-II infection also induces the formation of viroplasms, with NS79 being the key protein responsible for GCRV-II viroplasm formation. GCRV-II viroplasms are membranous structures that lack liquid-like properties. Notably, we confirmed that GCRV-II infection induces autophagy and utilizes autophagosomes for viroplasm formation and virion assembly. Furthermore, we found that GCRV-II utilizes autophagosomes for subclinical persistent infection, nonlytic release, and spread. Our results revealed the characteristics of GCRV-II viroplasms are significantly different from those of known animal reovirus, providing valuable information for the prevention and control of this virus.

## RESULTS

### GCRV-II infection induces the formation of viroplasms

**Fig 1:**
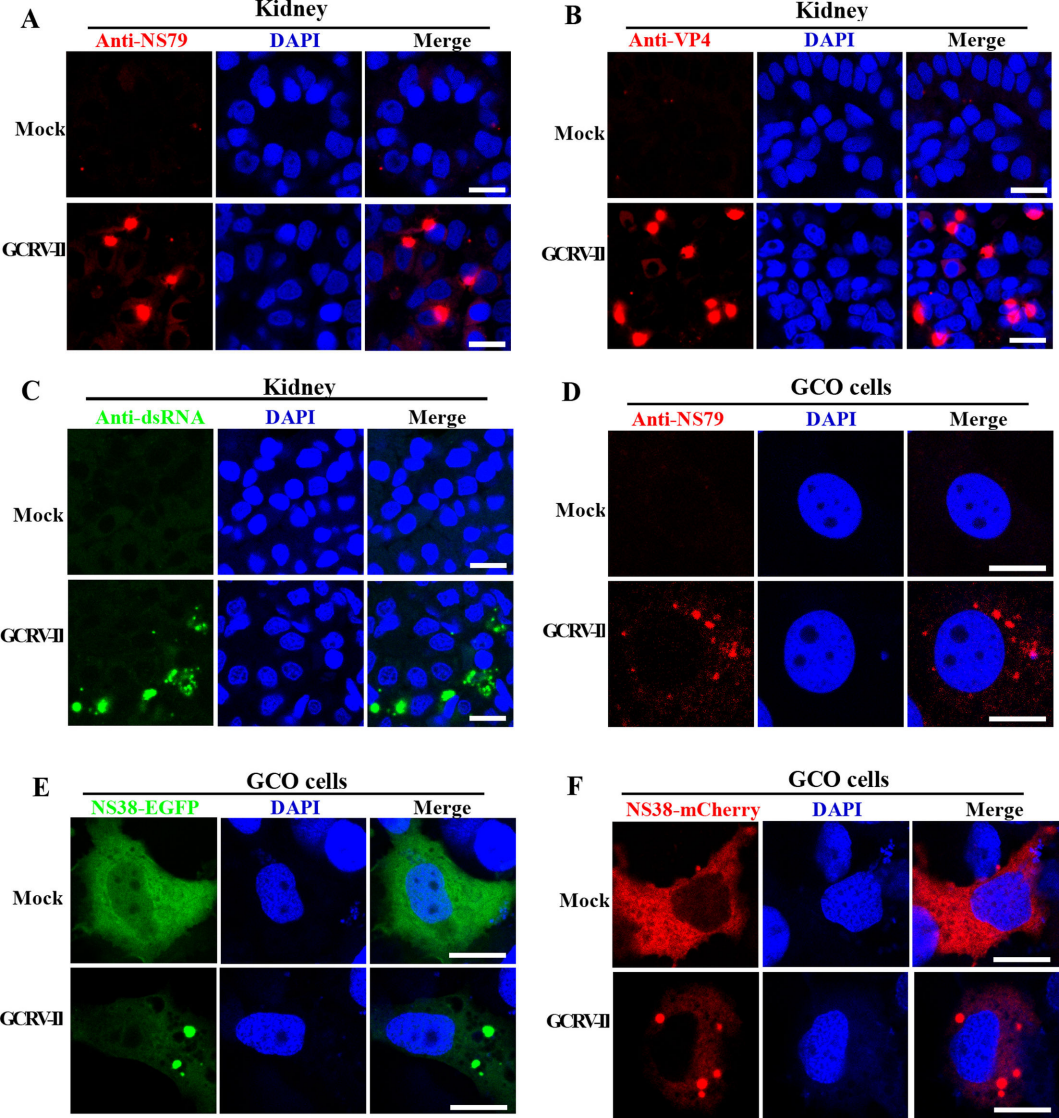
GCRV-II infection induces the formation of viroplasms. (**A–C**) Immunofluorescence analysis of kidney samples from GCRV-II-infected fish by using antibodies against N79 (**A**), VP4 (**B**), or dsRNA (**C**), respectively. Grass carp were mock-infected or infected with GCRV-II, and the kidney samples were collected for immunofluorescence analysis. Scale bar = 10 µm. (**D**) Immunofluorescence analysis of GCRV-II GCO cells using antibodies against NS79. GCO cells were mock-infected or infected with GCRV-II and then stained with anti-NS79 antibody. Scale bar = 10 µm. (**E, F**) The localization patterns of NS38-EGFP (**E**) and NS38-mCherry (**F**) in the absence or presence of GCRV-II infection. GCO cells were transfected with NS38-EGFP or NS38-mCherry plasmids and then mock-infected or infected with GCRV-II and harvested for fluorescence observation. Scale bar = 10 µm.

As the predominant strain of grass carp hemorrhagic disease virus currently circulating in China (4, 8, 9), the detailed molecular events regarding GCRV-II infection and viroplasm formation are still largely unknown. Therefore, grass carp were mock-infected or infected with GCRV-II, and the kidney and intestine samples were collected for immunofluorescence analysis. The viroplasms of reovirus are believed to contain viral proteins and viral RNA that participate in viral replication and assembly. Hence, we detected the viroplasms in GCRV-II-infected fish or control fish by using antibodies against nonstructural protein NS79, capsid protein VP4, and a speciﬁc antibody for double-stranded RNA (dsRNA). As shown in Fig. 1A through C, globular or punctate structures were observed in the cytoplasm of GCRV-II-infected kidney samples when stained with these antibodies. However, no such structures were found in control fish. These structures were similar to the viroplasms observed in MRV-, ARV-, bluetongue virus (BTV)-, or GCRV-I-infected cells (14, 17, 22, 23). Similar phenomena were also observed in intestine samples collected from GCRV-II-infected intestine samples but not in control intestine samples (Fig. S1A through C). To further investigate whether GCRV-II infection induces viroplasms *in vitro*, GCO cells were mock-infected or infected with GCRV-II and then collected for immunofluorescence by using antibodies against NS79 or VP4. Similarly, we also observed globular or punctate structures in the cytoplasm of GCRV-II-infected GCO cells but not in the mock-infected cells (Fig. 1D; Fig. S1D). Furthermore, previous studies suggested that nonstructural protein NS38 is involved in GCRV-II viroplasm formation (4, 8, 9). Therefore, GCO cells were transfected with EGFP- or mCherry-tagged NS38 and then mock-infected or infected with GCRV-II. As shown in Fig. 1E and F, both the NS38-EGFP and NS38-mCherry diffusely distributed in the cytoplasm of GCO cells in the absence of GCRV-II infection, while formed globular structures, referred to as viroplasm-like structures (VLSs), in the presence of GCRV-II infection. Collectively, these results strongly imply that GCRV-II infection induces the formation of viroplasms.

### NS79 is responsible for GCRV-II viroplasm formation

**Fig 2:**
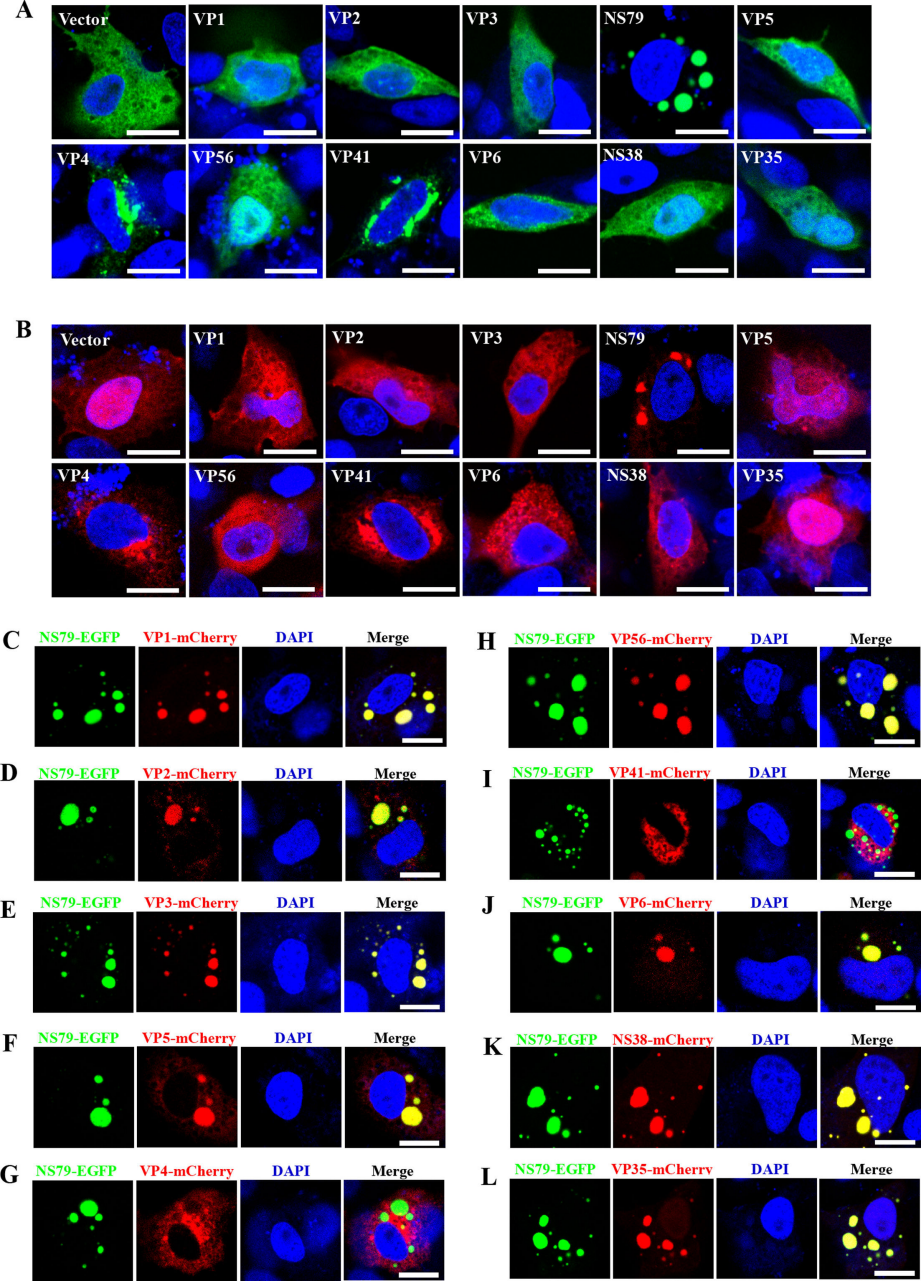
NS79 is responsible for GCRV-II viroplasm formation. (**A, B**) The localization patterns of all GCRV-II-encoded proteins. GCO cells were transfected with 11 EGFP-tagged (**A**) or mCherry-tagged (**B**) viral proteins or empty vector and harvested at 24 hours post-infection for fluorescence observation. Scale bar = 10 µm. (**C–L**) The relationship between viral NS79-EGFP and other mCherry-tagged viral proteins. NS79-EGFP was co-transfected with other mCherry-tagged viral proteins into GCO cells and harvested at 24 hours post-infection for fluorescence observation. Scale bar = 10 µm.

We therefore investigated which viral-encoded protein was responsible for GCRV-II viroplasm formation. The nonstructural protein NS79 has received special attention due to its being a homolog of NS80 in GCRV-I and µNS in MRV and ARV. NS80 and µNS are key proteins for viroplasm formation and are known to form VLSs even in the absence of virus infection (14, 16, 17, 21). Upon transfecting EGFP-tagged viral proteins into cells, we found NS79-EGFP formed globular inclusion structures that are similar to viroplasms, referred to as VLSs, *in vitro* (Fig. 2A). Interestingly, we also found that VP4-EGFP and VP41-EGFP formed irregular aggregated structures at the periphery areas of the nucleus. In contrast, the remaining proteins were uniformly distributed in the cytoplasm or throughout the entire cells (Fig. 2A). Similar localization patterns were also observed when these viral proteins were fused with the mCherry tag (Fig. 2B). After that, we investigated the relationship between NS79 and other viral encoded proteins. NS79-EGFP were co-transfected with other mCherry-tagged viral-encoded proteins and then analyzed by confocal microscopy. As shown in Fig. 2C through L, for the ten examined proteins, most of them co-aggregated with the VLSs formed by NS79-EGFP, except for VP4 and VP41 (Fig. 2G and I), which still formed irregular aggregated structures at the periphery areas of the nucleus. Given that NS79 is the homolog of NS80 and µNS, and that it forms VLSs *in vitro*, as well as recruits most of the other viral-encoded proteins into VLSs, it is convincing that NS79 is responsible for the formation of GCRV-II viroplasms.

### GCRV-II viroplasms lack liquid-like properties

**Fig 3:**
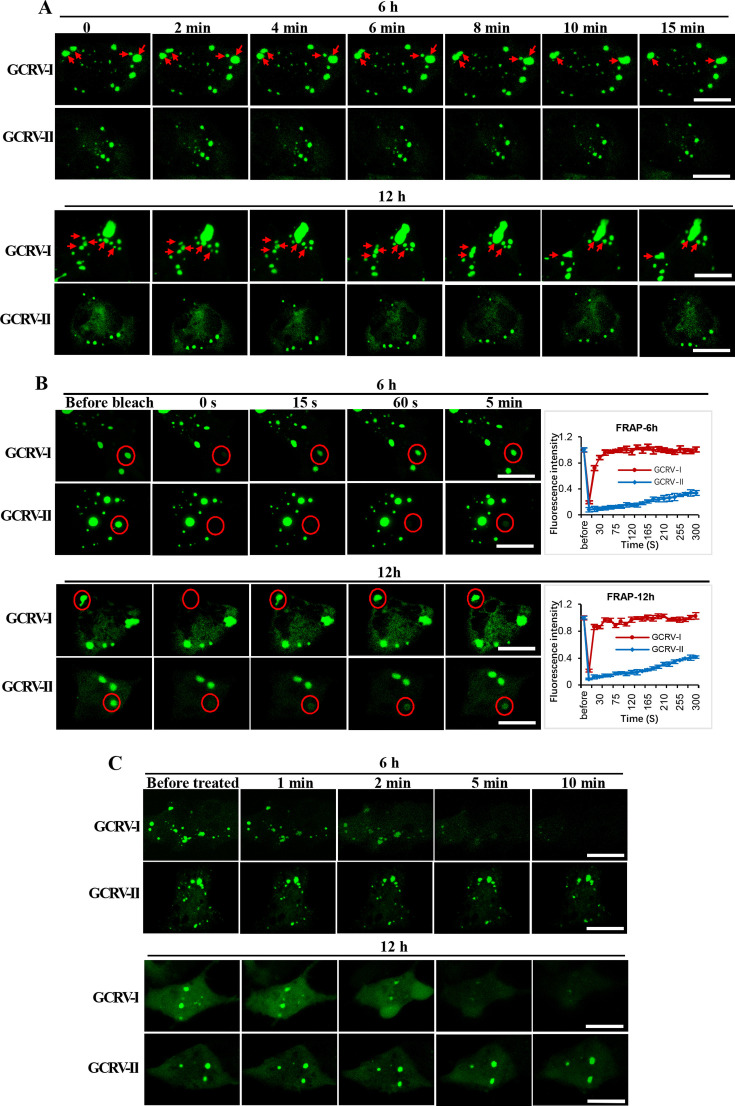
GCRV-II viroplasms lack liquid-like properties. (**A**) Investigate the movement of GCRV-I and GCRV-II viroplasms by live-cell imaging. GCO cells were transfected with NS38-EGFP of GCRV-I or GCRV-II and then infected with the corresponding virus and collected at 6 or 12 hours post-infection for live-cell imaging analysis. The red arrows indicate the small viroplasm puncta fusing and merging into larger puncta. Scale bar = 10 µm. (**B**) FRAP assay of GCRV-I and GCRV-II viroplasms. GCO cells were transfected with NS38-EGFP of GCRV-I or GCRV-II and then infected with the corresponding virus and collected at 6 or 12 hours post-infection for FRAP assay. Data are represented as mean (*n* = 3) ±SD. Scale bar = 10 µm. (**C**) Investigate the liquid-like properties of GCRV-I and GCRV-II viroplasms by 1,6-HD treatment. GCO cells were transfected with NS38-EGFP of GCRV-I or GCRV-II and then infected with the corresponding virus and collected at 6 or 12 hours post-infection. Scale bar = 10 µm.

Previously, studies revealed that reovirus viroplasms are highly dynamic structures with liquid-like properties (18, 19, 21). We therefore investigate the liquid-like properties of GCRV-II viroplasms by live-cell imaging. Due to the lack of a fluorescence-tagged GCRV to monitor the viroplasms, we chose the nonstructural protein NS38 as the marker for GCRV-II viroplasms. As EGFP or mCherry tagged, NS38 diffusely distributed in the cytoplasm of transfected cells in the absence of GCRV-II infection while forming VLSs in the presence of GCRV-II infection. Therefore, GCO cells were transfected with NS38-EGFP and infected with GCRV-II, and cells were harvested at 6 and 12 hours post-infection (hpi) to investigate the movement of GCRV-II viroplasms. Cells were imaged every 15 seconds within an approximate 15 minute period, and a movie was created. As depicted in Fig. 3A, we observed no fusion and fission events occurring during the 15 minute period for the GCRV-II viroplasms at 6 and 12 hpi. As a positive control, a total of two and four fusion events were detected in GCRV-I viroplasms at the same time points (Fig. 3A). To quantify this observation, we selected 15 cells for observation and analyzed the number of fusion and fission events. Figure S2A and B showed that the fusion and fission events occurred frequently in GCRV-I viroplasm, whereas they were hard to detect in GCRV-II viroplasms at 6 and 12 hpi.

We also performed fluorescence recovery after the photobleaching (FRAP) assay to further investigate the liquid properties of GCRV-II viroplasms. The NS38-EGFP-transfected and GCRV-II-infected cells were harvested at 6 and 12 hpi. Regions of interest (ROIs) in the viroplasms (indicated by NS38-EGFP) were photobleached, and the recovery of fluorescence was monitored over time. The FRAP assay for GCRV-I viroplasm served as a positive control. Consistent with our previous study (21), the fluorescence signal of the ROIs in GCRV-I viroplasms rapidly recurred within just 15 seconds post-bleaching and fully recovered to nearly 100% of the original intensity in less than 1 minute at 6 and 12 hpi (Fig. 3B), further implying the liquid-like properties of GCRV-I viroplasms. However, the fluorescence signal from ROIs in GCRV-II viroplasms recovered no more than 40% of the initial intensity within 5 minutes, indicating that GCRV-II viroplasms lack liquid-like properties.

Moreover, we also treated GCRV-II-infected cells with 4% 1,6-hexanediol (1,6-HD), a commonly used chemical probe that dissolves LLPS assemblies (18), to further investigate the liquid-like properties of GCRV-II viroplasms. As shown in Fig. 3C, the fluorescence signal of GCRV-II viroplasm puncta at 6 and 12 hpi was unaffected after the addition of 1,6-HD to the cell culture medium and even persistent for more than 10 minutes of treatment. However, as a positive control, we observed that the fluorescence signal of GCRV-I viroplasms puncta at 6 and 12 hpi significantly decreased after the addition of 1,6-HD to the cell culture medium and almost completely dissolved after a 10 minute treatment (Fig. 3C).

Furthermore, we also investigated whether NS79, the protein that is responsible for GCRV-II viroplasm formation, possesses LLPS properties by transfecting the NS79-EGFP plasmid into cells. Live-cell imaging showed that no fusion and fission events occurred during a 15 minute period after 6 and 12 hours post-transfection (Fig. S2C). The FRAP assay revealed that the fluorescence signal of NS79 puncta was hard to recover post-bleach (Fig. S2D). Moreover, we also found that NS79 puncta were resistant to 1,6-HD treatment (Fig. S2E). Taken together, the results demonstrate that GCRV-II viroplasms lack liquid-like properties.

### GCRV-II viroplasms are bound by membranes

**Fig 4:**
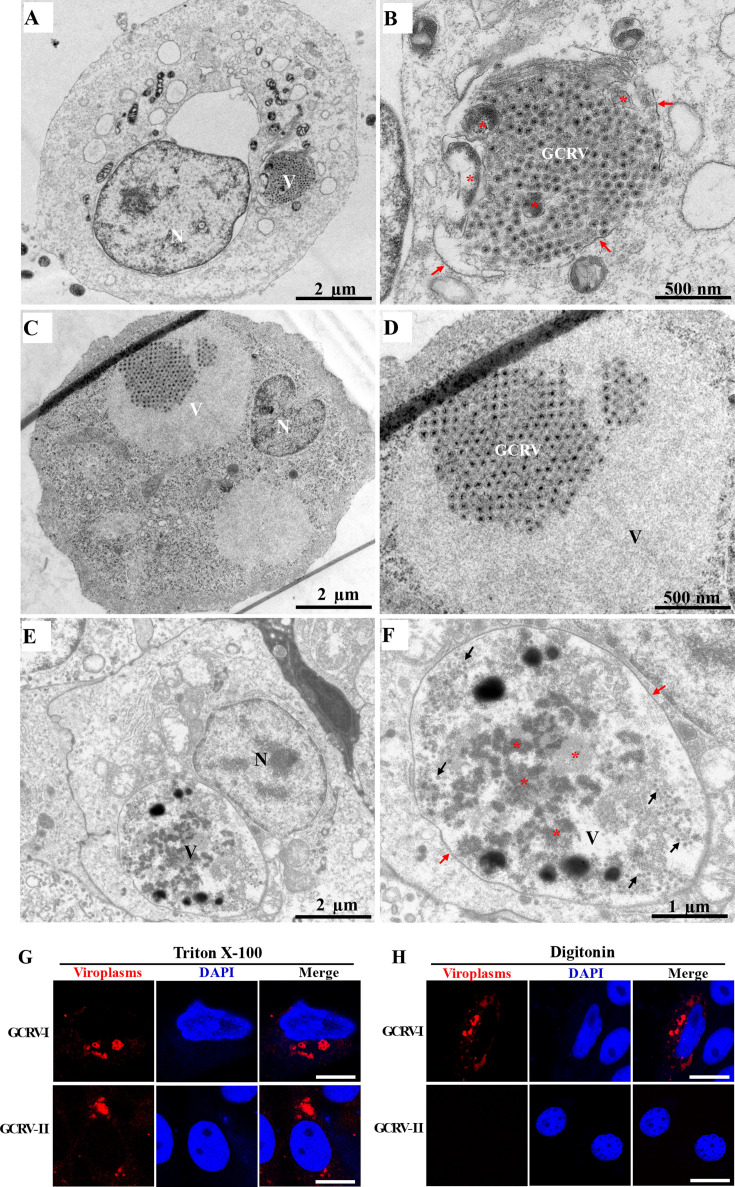
GCRV-II viroplasms are bound by membranes. (**A, B**) TEM analysis of GCRV-II-infected cells. The red arrows indicate the membranes of viroplasms, while the asterisks indicate the cytosolic components or organelles. N: nucleus; V: viroplasm; GCRV: the GCRV virions. Scale bar = 2 µm and 500 nm. (**C, D**) TEM analysis of GCRV-I-infected cells. V: viroplasm; GCRV: the GCRV virions. Scale bar = 2 µm and 500 nm. (**E, F**) TEM analysis of kidney samples from GCRV-II-infected fish. N: nucleus; V: viroplasm. The red arrows indicate the membranes of viroplasms, the asterisks indicate the cytosolic components or organelles, and the black arrows represent the GCRV-II virions. Scale bar = 2 µm and 1 µm. (**G, H**) Immunofluorescence analysis of GCRV-I- or GCRV-II-infected cells that permeabilized with either Triton X-100 (**G**) or digitonin (**H**). GCO cells were infected with GCRV-I or GCRV-II and then harvested at 24 hpi. Cells permeabilized with either Triton X-100 (**G**) or digitonin (**H**) and then stained with anti-NS80 or anti-NS79 antibodies to detect viroplasms. Scale bar = 10 µm.

Another characteristic of reovirus viroplasms is that they are membraneless structures, which are essential for their liquid-like properties (21). However, it remains unknown whether the viroplasms of GCRV-II are also membraneless structures. Therefore, GCO cells were infected with GCRV-I or GCRV-II and harvested at 48 hpi for TEM analysis. As depicted in Fig. 4A and B, the TEM results showed that the GCRV-II viroplasms are clearly bound by membranes, in which the GCRV-II virions arranged orderly. Interestingly, we also found some cytosolic components or organelles along with the GCRV-II virions sequestered by the membranes. As a control, the GCRV-I viroplasms are membraneless structures that significantly distinguish from other membrane-bound cell organelles, in which the GCRV-I virions are also arranged orderly (Fig. 4C and D). Nevertheless, no cytosolic components or organelles were found in GCRV-I viroplasms (Fig. 4C and D). Moreover, grass carp were infected with GCRV-II, and the kidney samples were collected for TEM analysis. As shown in Fig. 4E and F, we clearly observed that the GCRV-II viroplasms were completely enclosed by membranes, in which the GCRV-II virions and cytosolic components or organelles were sequestered.

To further investigate whether the GCRV-II viroplasms are bound by membranes, GCO cells were infected with GCRV-I or GCRV-II and then harvested at 24 hpi. Cells were fixed and permeabilized using either Triton X-100 or digitonin. The former permeabilizes both the plasma membrane and internal membranes, whereas the latter only permeabilizes the plasma membrane, but not intracellular membranes (24). Subsequently, cells were stained with anti-NS80 or anti-NS79 antibodies to detect viroplasms from GCRV-I or GCRV-II, respectively. As shown in Fig. 4G and H, the GCRV-I viroplasms were visible following either permeabilization method. However, the GCRV-II viroplasms were only visible in Triton X-100-permeabilized cells, whereas it was hard to detect in digitonin-permeabilized cells (Fig. 4G and H). Collectively, these results strongly confirm that GCRV-II viroplasms are bound by membranes that significantly distinguish from GCRV-I viroplasms.

### GCRV-II infection induces autophagy and autophagosome formation

**Fig 5:**
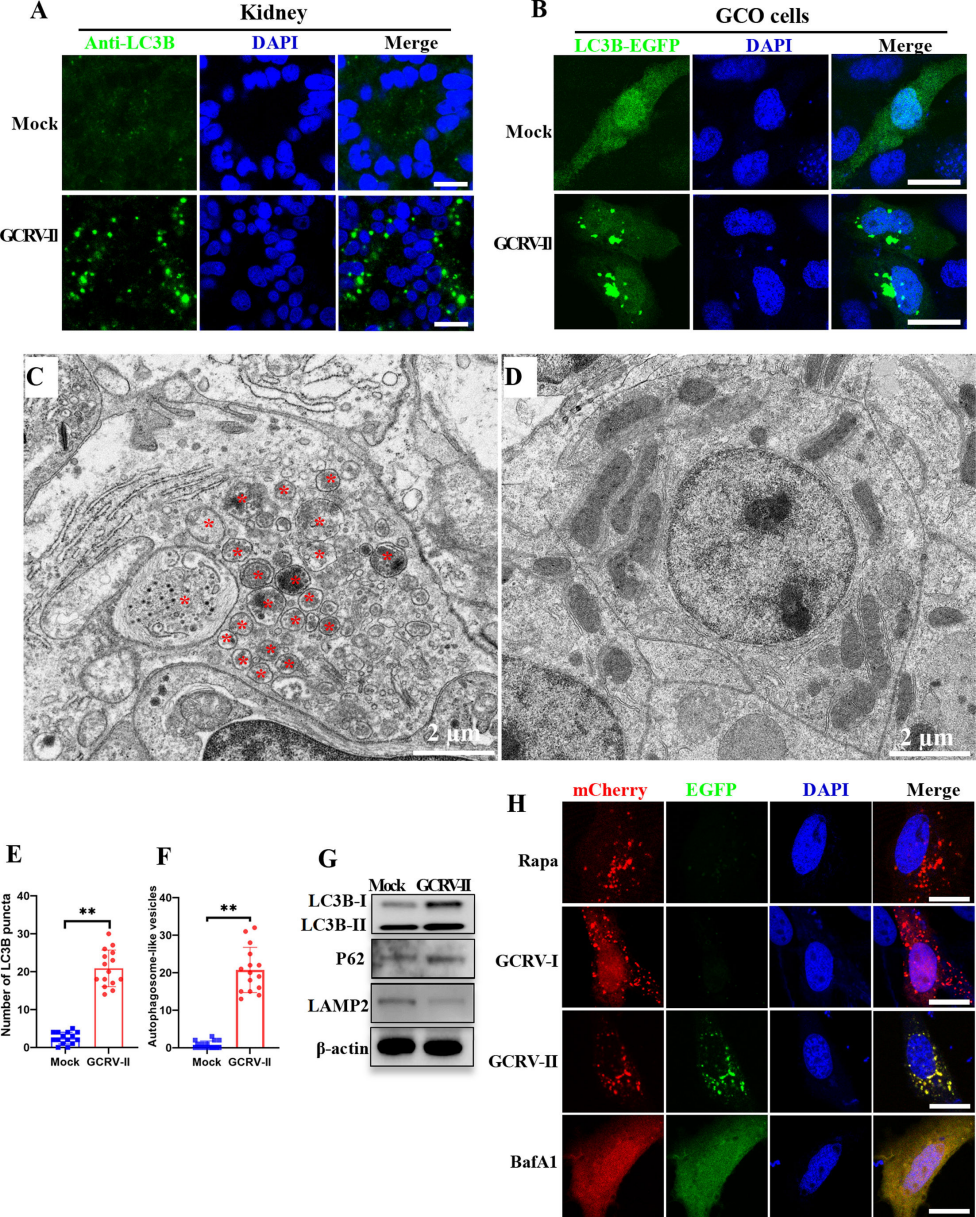
GCRV-II infection induces autophagy and autophagosome formation. (**A**) Immunofluorescence analysis of kidney samples from GCRV-II-infected or control fish by using antibodies against LC3B. Scale bar = 10 µm. (**B**) The localization patterns of LC3B-EGFP in the absence or presence of GCRV-II infection. Scale bar = 10 µm. (**C, D**) TEM analysis of kidney samples from GCRV-II-infected or control fish. The asterisks indicate autophagosome-like vesicles in GCRV-infected kidney samples. Scale bar = 2 µm. (**E**) Quantitative analysis of the number of LC3B puncta in mock- or GCRV-II-infected cells. Data are represented as mean (*n* = 15) ±SD. ** indicates *P* < 0.01. (**F**) Quantitative analysis of the number of autophagosome-like vesicles in GCRV-II-infected or control fish. Data are represented as mean (*n* = 15) ±SD. ** indicates *P* < 0.01. (**G**) Western blotting analysis of the protein expression level of LC3B, P62, and LAMP2 in kidney samples from GCRV-II-infected or control fish. (**H**) Investigation of the process of autophagic flux during GCRV-II infection by using the pCMV-mCherry-GFP-LC3B plasmid. Cells were transfected with this plasmid and then treated with the autophagy inducer rapamycin (Rapa) or the autophagy inhibitor bafilomycin A1 (BafA1) or infected with GCRV-I or GCRV-II. Cells were harvested at 24 hours for fluorescence observation. Scale bar = 10 µm.

The above-mentioned results indicated that GCRV-II viroplasms are enclosed by membranes. Moreover, we also found GCRV virions, as well as cytosolic components or organelles, were sequestered by these membranes. The virion-contained membranous vesicles are morphologically similar to autophagosomes. Therefore, we investigate whether GCRV-II infection induces autophagy and autophagosome formation. Grass carp infected with GCRV-II and the intestine and kidney samples were collected for analysis. RT-qPCR showed that expression levels of numerous autophagy-related genes (ATG5, ATG10, ATG12, LC3B, Beclin-1, and P62) were significantly increased in GCRV-infected fish when compared with control fish (Fig. S3A through F). The hallmark of autophagy is the redistribution of LC3B from a diffuse localization to a characteristic punctate pattern, implying the recruitment of LC3B to autophagosomes (25, 26). Therefore, immunofluorescence was performed for kidney and intestine samples collected from GCRV-infected fish by using the LC3B antibody. As shown in Fig. 5A and Fig. S3G, the LC3B formed as a punctate pattern in GCRV-II-infected samples, whereas uniformly distributed in control fish. We also transfected EGFP- or mCherry-tagged LC3B into GCO cells and then mock-infected or infected with GCRV-II. As depicted in Fig. 5B and Fig. S3H, the fluorescence of EGFP-LC3B or mCherry-LC3B was diffusely distributed throughout the mock-infected cells, whereas formed as a punctate pattern in GCRV-II-infected cells. Furthermore, we performed TEM analysis for the kidney samples that were collected from GCRV-II-infected or mock-infected fish. As expected, a large number of autophagosome-like vesicles were observed in the sample from GCRV-infected fish but hard to detect in the mock-infected fish (Fig. 5C and D; Fig. S3I and J). Importantly, we also observed that many GCRV virions were enclosed by such autophagosome-like vesicles (Fig. 5C; Fig. S3I). Quantitative analysis showed the average number of LC3B puncta and autophagosome-like vesicles in the GCRV-II-infected group was remarkably higher than that of the mock-infected group (Fig. 5E and F). Taken together, these results indicate GCRV-II infection induces autophagy and autophagosome formation.

Moreover, we also investigated the progress of autophagic flux during GCRV-II infection. Autophagic adapter SQSTM1/p62 (sequestosome 1) is an indicator to assess autophagic flux because SQSTM1 can target specific cargo for autophagy and is specifically degraded by the autophagic–lysosomal pathway (27). Western blotting indicated that the protein level of LC3B-II was increased notably in GCRV-II-infected fish, further suggesting the activation of autophagy after GCRV-II infection (Fig. 5G). However, the protein level of p62 was not decreased after GCRV-II infection (Fig. 5G). Moreover, we also found the expression level of lysosome-associated membrane protein 2 (Lamp2) was not increased after GCRV-II infection (Fig. 5G). We further investigated autophagic flux during GCRV-II infection using the pCMV-mCherry-GFP-LC3B plasmid, a reliable marker for autophagic flux, as it allows measurement of autophagosome–lysosome fusion (28). Cells were transfected with the plasmid and treated with the autophagy inducer rapamycin (Rapa), the autophagy inhibitor bafilomycin A1 (BafA1), or infected with GCRV-I or GCRV-II. After 24 hours, fluorescence was observed to detect GFP and mCherry puncta. When autophagosomes fuse with lysosomes, GFP puncta are rapidly degraded by lysosomes, while mCherry puncta remain intact, allowing only mCherry signals to be detected. Blocking autophagosome–lysosome fusion prevents GFP degradation, so both GFP and mCherry signals are visible. As shown in Fig. 5H, in the Rapa-treated or GCRV-I-infected cells, only mCherry puncta were detected, with few GFP puncta observed, indicating fusion of autophagosomes with lysosomes. In BafA1-treated cells, both mCherry and GFP were distributed uniformly across the cell, suggesting autophagy inhibition. In GCRV-II-infected cells, both GFP and mCherry puncta signals were observed, indicating a block in autophagosome–lysosome fusion. Therefore, these results indicate that autophagy was activated, while autophagic flux was inhibited after GCRV-II infection.

### GCRV-II utilizes autophagosome for viroplasm formation and virion assembly

**Fig 6:**
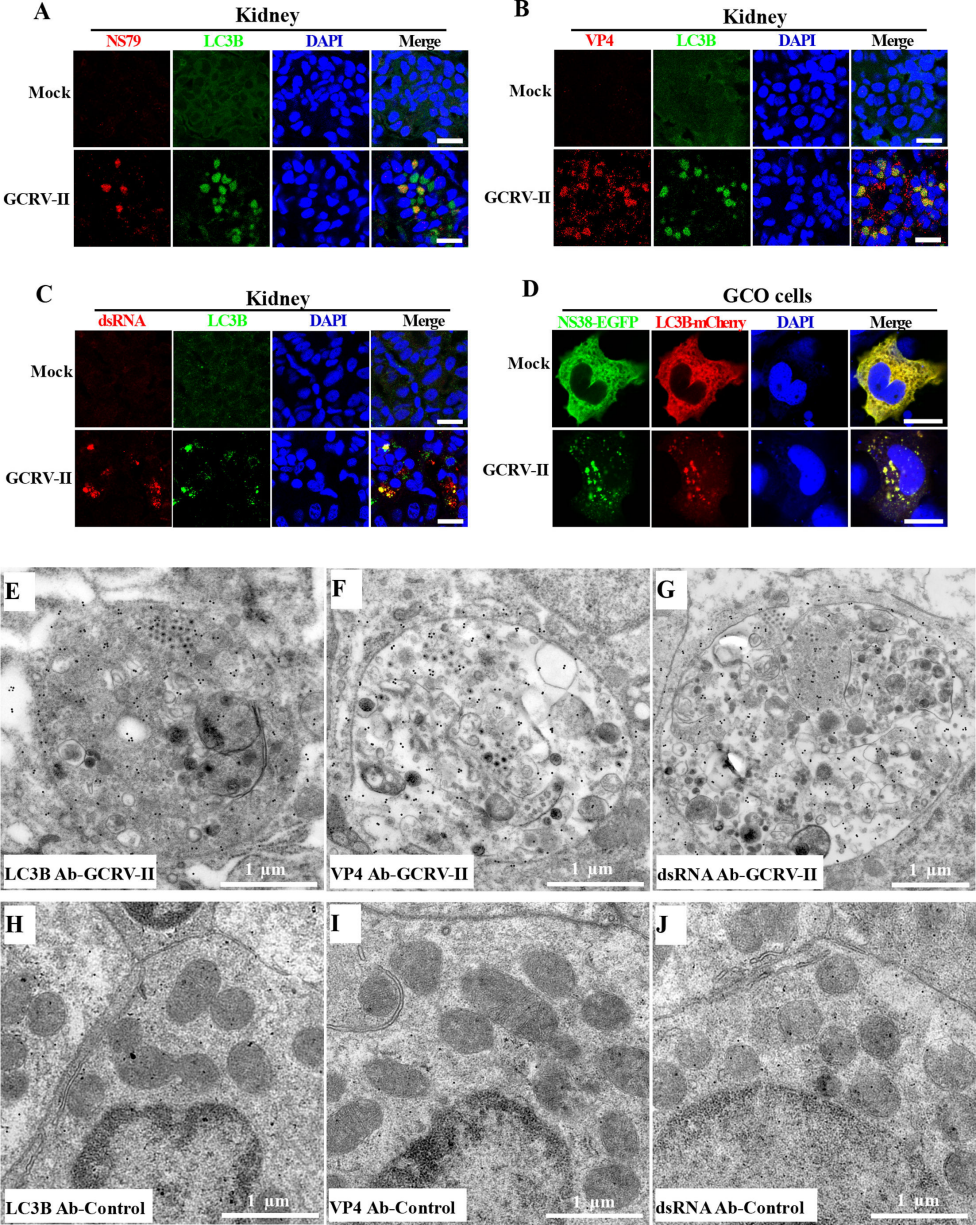
GCRV-II utilizes autophagosomes for viroplasm formation and virion assembly. (**A–C**) Immunofluorescence analysis of the relation between GCRV-II viroplasms and autophagosomes. Grass carp were mock-infected or infected with GCRV-II, and the kidney samples were collected. The autophagosomes were stained with the anti-LC3B antibody, while the viroplasms were stained with anti-NS79, anti-VP4, or anti-dsRNA antibodies. Scale bar = 10 µm. (**D**) The relationship between viral NS38-EGFP and LC3B-mCherry in the absence or presence of GCRV-II infection. Scale bar = 10 µm. (**E–G**) Immunoelectron microscopy analysis of kidney samples from GCRV-II-infected fish. The GCRV-II-infected kidney samples were immunolabeled with LC3B (**E**), VP4 (**F**), or ds RNA (**G**) antibodies as the primary antibody, followed by treatment with 10 nm gold particle-conjugated IgG as the secondary antibody. Scale bar = 1 µm. (**H–J**) Immunoelectron microscopy analysis of kidney samples from control fish. The control kidney samples were immunolabeled with LC3B (**H**), VP4 (**I**), or ds RNA (**J**) antibodies as the primary antibody, followed by treatment with 10 nm gold particle-conjugated IgG as the secondary antibody. Scale bar = 1 µm.

We therefore investigate whether GCRV-II utilizes virus-induced autophagosomes for viroplasm formation and virion assembly. Immunofluorescence was performed to investigate the relationship between viroplasms and autophagosomes by using LC3B and GCRV-II NS79 or VP4 antibodies. Figure 6 clearly revealed that NS79- or VP4 antibody-stained viroplasms colocalized with the LC3B-stained autophagosomes (Fig. 6A and B). Furthermore, when the viroplasms stained with the dsRNA antibody, similar colocalization patterns were also observed (Fig. 6C). Moreover, NS38-EGFP was co-transfected with LC3B-mCherry into GCO cells and then mock-infected or infected with GCRV and harvested for analysis. Consistent with the result above, the NS38-EGFP formed VLSs in the presence of GCRV-II infection while diffusely distributed in the cytoplasm in the absence of GCRV infection. Importantly, we clearly observed that LC3B-mCherry-tagged autophagosomes co-aggregated with NS38-EGFP-formed VLSs in the presence of GCRV-II infection (Fig. 6D). To further confirm that the virion-contained membranous vesicles are indeed autophagosomes, we performed immunoelectron microscopy (IM) for the GCRV-infected kidney samples. IM showed that the virion-contained membranous vesicles could be specifically labeled by LC3B antibodies, suggesting these vesicles are indeed autophagosomes (Fig. 6E). Moreover, we also found that such virion-contained membranous vesicles reacted with the GCRV-II VP4 antibody and dsRNA antibody (Fig. 6F and G), indicating GCRV-II replication and assembly in the autophagosomes. Nevertheless, the control kidney samples showed no reaction with any of the three antibodies, as evidenced by the absence (or near absence) of immunogold particles in these samples (Fig. 6H through J). Collectively, these results suggest that GCRV-II utilizes autophagosomes for viroplasm formation and virion assembly.

### GCRV-II utilizes autophagosomes for subclinical persistent infection

**Fig 7:**
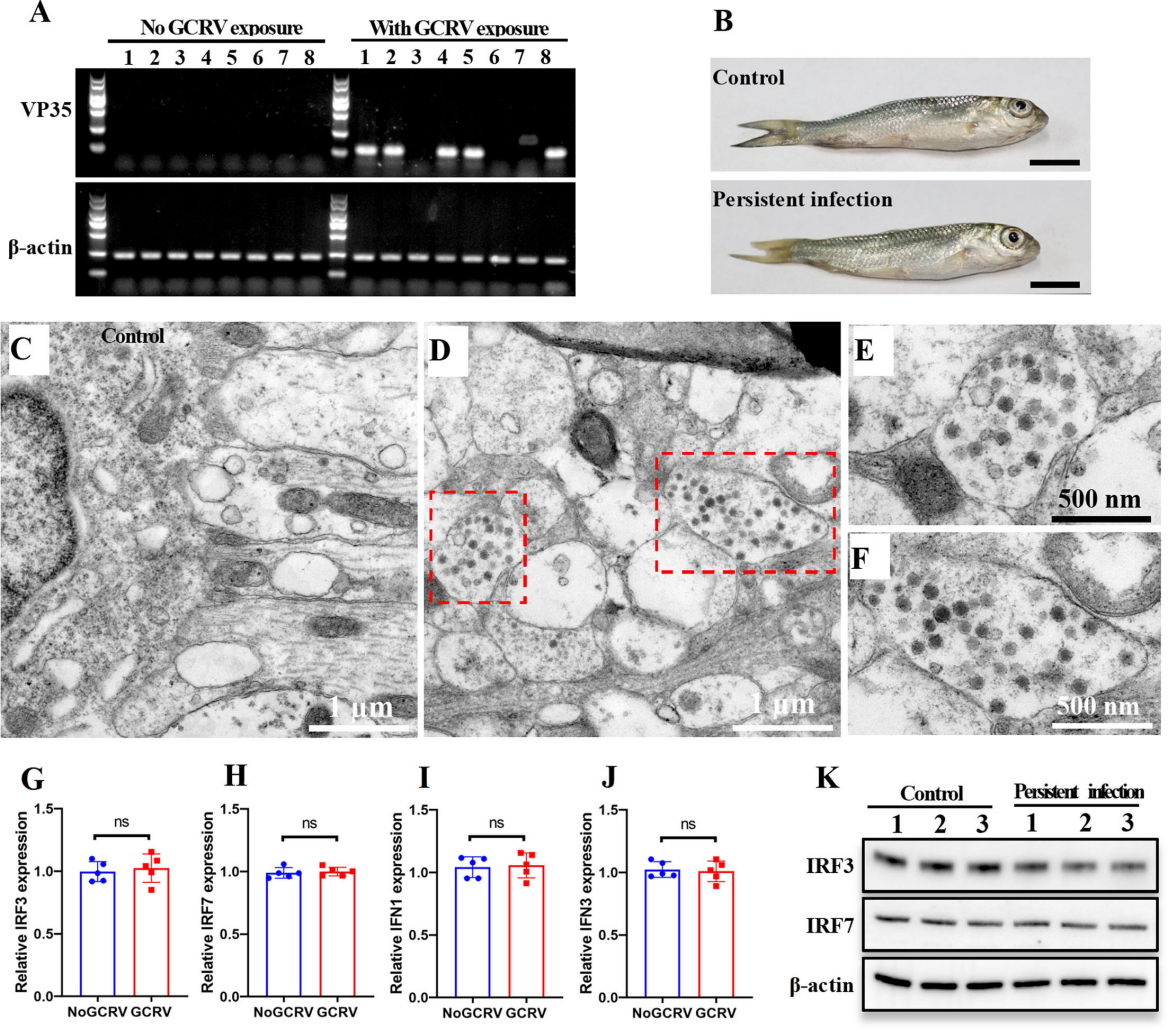
GCRV-II utilizes autophagosomes for subclinical persistent infection. (**A**) RT-PCR detection of GCRV-II in grass carp from fish farms with or without GCRV exposure history. Eight individuals were collected from each farm for GCRV-II detection by using primers specific for VP35, and β-actin was used as an internal control. (**B**) Clinical observation of GCRV-II subclinical persistent infected fish and control fish. Scale bar = 1 cm. (**C, D**) TEM analysis of brain samples from control fish (**C**) or from GCRV-II subclinical persistent infected fish (**D**). The red boxes indicate the virions that are enclosed by autophagosome-like vesicles. Scale bar = 1 µm. (**E, F**) Magnification of virion-contained autophagosome-like vesicles in GCRV-II subclinical persistent infected fish. Scale bar = 500 nm. (**G–J**) RT-qPCR analysis of the mRNA expression levels of IRF3 (**G**), IRF7 (**H**), IFN1 (**I**), and IFN3 (**J**) in GCRV-II subclinical persistent infected fish and control fish. Data are represented as mean (*n* = 5) ±SD. ns indicates no significant difference. (**K**) Western blotting analysis of the protein expression levels of IRF3 and IRF7 in three GCRV-II subclinical persistent infected fish and in three control fish.

A previous study suggested that GCRV-II could establish latent infection, also known as subclinical persistent infection, in brain tissues of grass carp (29). Therefore, we collected grass carp from fish farms with or without GCRV exposure history to further investigate the molecular mechanism of subclinical persistent infection. The kidney and brain tissues of these fish were removed for analysis. RT-PCR revealed that a specific viral band was amplified in some fish with a GCRV-II exposure history, but not in fish without GCRV-II exposure history (Fig. 7A), suggesting the presence of subclinical persistent GCRV-II infection. Moreover, no hemorrhagic symptoms were observed in body surfaces of subclinical persistent infected and control fish (Fig. 7B). Histological section analysis also showed that no pathological changes were observed in the kidney samples from both fish (Fig. S4A and B). However, some pathological changes, such as loose brain matrix and cell necrosis, were observed in the brain tissues of subclinical persistent infected fish, but not in the control fish (Fig. S4C and D). TEM analysis showed numerous GCRV-II virions were observed in brain tissues from the subclinical persistent infected fish, but none in the control fish (Fig. 7C and D). Importantly, these virions were also enclosed by autophagosome-like vesicles (Fig. 7E and F), indicating that GCRV-II utilizes autophagosomes for subclinical persistent infection. The hiding of virions within autophagosomes may be a strategy to evade the host immune system. To further prove this hypothesis, we analyzed the expression levels of interferon-related genes between two fish groups. As expected, RT-qPCR revealed no significant differences in the mRNA expression levels of interferon regulatory factors (IRF3 and IRF7) (Fig. 7G and H) and interferons (IFN1 and IFN3) (Fig. 7I and J) between the two groups. Moreover, Western blotting analysis also showed similar protein expression patterns of IRF3 and IRF7 between the two groups (Fig. 7K). Collectively, these results strongly suggest that GCRV-II utilizes autophagosomes to establish subclinical persistent infection and evade the host immune system.

### GCRV-II utilizes autophagosomes for nonlytic release and spread

**Fig 8:**
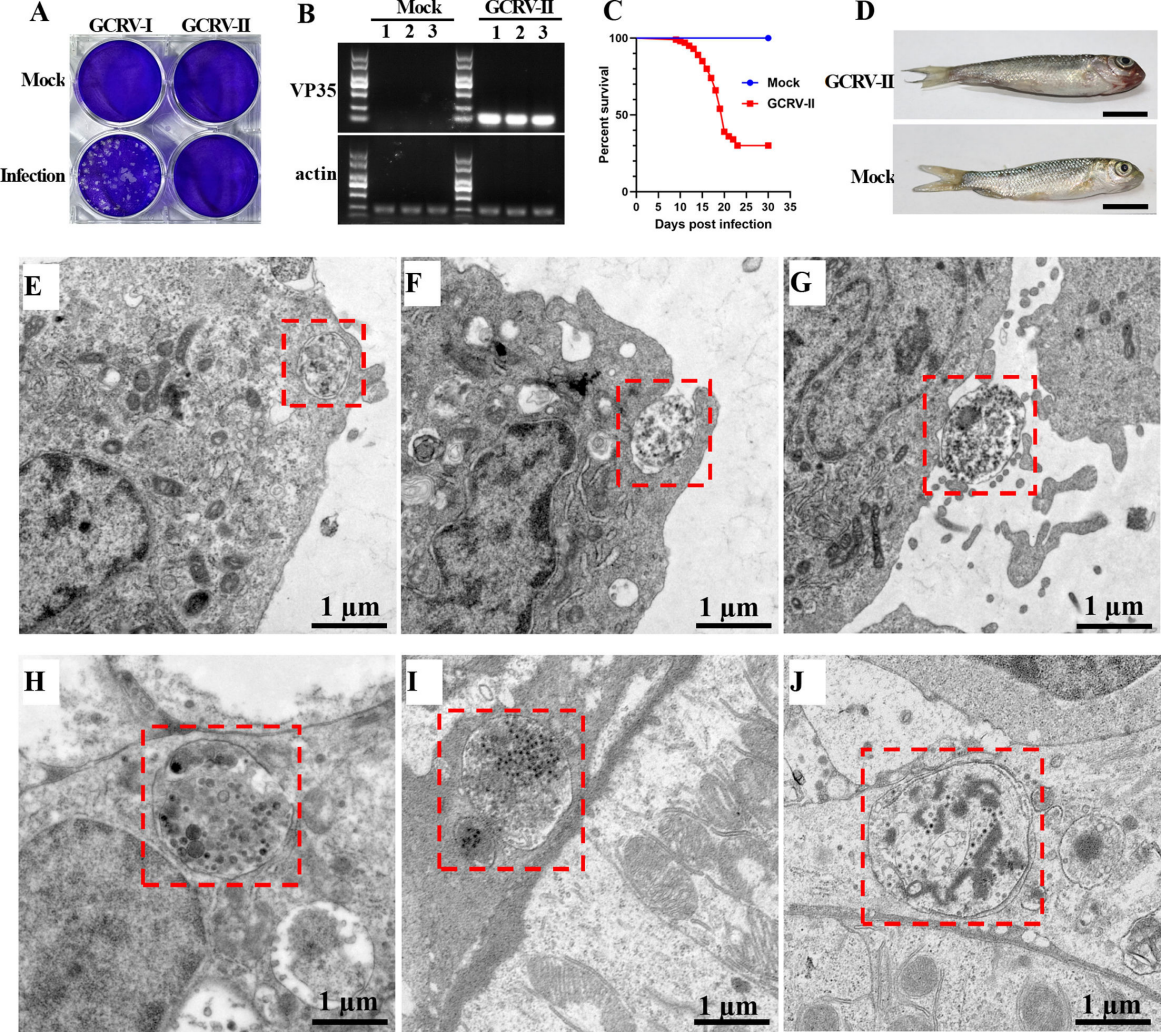
GCRV-II utilizes autophagosomes for non-lytic release and spread. (**A**) GCRV-II infection did not induce CPE in CIK cells. CIK cells were mock-infected or infected with GCRV-I or GCRV-II and then stained by crystal violet. (**B**) RT-PCR detection of GCRV-II in mock- or GCRV-II-infected cells. GCRV-II detection was performed by using primers specific for VP35, and β-actin was used as an internal control. (**C**) Percent of survival in grass carp that were injected with supernatants from GCRV-II-infected or mock-infected cells. (**D**) Clinical observation of grass carp that were injected with supernatants from GCRV-II (upper)- or mock-infected cells (bottom). Scale bar = 1 cm. (**E–G**) TEM analysis of GCRV-II-infected cells that were harvested at different time points. The virion-contained autophagosomes were observed at the periphery of cell membranes (**E**), fused with the cytomembrane (**F**), or outside the cells (**G**). Scale bar = 1 µm. (**H–J**) TEM analysis of kidney samples from GCRV-II-infected fish that were collected at different time points. The virus-containing autophagosomes were observed at the periphery of kidney cell membranes (**H**), outside the cells (**I**), or located at the intercellular junctional regions between the kidney cells (**J**). Scale bar = 1 µm.

To further investigate the role of autophagosomes during GCRV-II infection, CIK cells were either mock-infected or infected with GCRV-I or GCRV-II. As expected, GCRV-I infection induced obvious CPE in CIK cells, whereas no CPE was observed in GCRV-II-infected cells (Fig. 8A). RT-PCR showed that a specific viral band was amplified in GCRV-II-infected cells but not in mock-infected cells, suggesting the success of GCRV-II infection (Fig. 8B). Moreover, the supernatant was collected from GCRV-II- or mock-infected cells and then used to infect grass carp. We found that the supernatant from GCRV-II-infected cells caused more than 70% fish mortality and induced typical hemorrhagic symptoms in grass carp, whereas the supernatant from mock-infected cells did not (Fig. 8C and D), implying the non-lytic release of progeny virus from GCRV-II-infected cells. Moreover, to test the role of autophagy in non-lytic release of GCRV-II, GCRV-II-infected cells were untreated or treated with the autophagy inducer rapamycin (Rapa) or the autophagy inhibitor 3-methyladenine (3-MA). The supernatants were collected, and the relative copy number of GCRV-II was measured. The results showed that treatment with Rapa increased the copy number of GCRV-II in the supernatants, while treatment with 3-MA resulted in the opposite trend (Fig. S4E and F). However, neither treatment promoted virus-induced cytopathic effect (CPE) in the cells (Fig. S4G), suggesting that autophagy is required for the nonlytic release of GCRV-II. To further investigate the detailed molecular events during GCRV-II infection, the GCRV-II-infected cells were harvested at different time points for TEM analysis. As shown in Fig. 8, TEM analysis revealed virion-containing autophagosomes at the periphery of cell membranes (Fig. 8E), fused with the cytomembranes (Fig. 8F), or outside the cells (Fig. 8G). It seemed that virion-containing autophagosomes can directly fuse with the plasma membrane to release viral particles. Thus, virion-containing autophagosomes may be involved in the non-lytic release of GCRV from culture cells, which may be the primary reason why it could not induce CPE in culture cells. Moreover, the kidney samples from GCRV-II-infected fish were also collected at different time points for TEM analysis. Similarly, we also observed virus-containing autophagosomes at the periphery of kidney cell membranes (Fig. 8H), outside the cells (Fig. 8I), or located at the intercellular junctional regions between the kidney cells (Fig. 8J). Therefore, it is likely that GCRV-II can exploit autophagosomes as the vehicles to overcome the membrane barriers and therefore facilitate viral spread.

## DISCUSSION

GCRV, a dsRNA virus belonging to the *Aquareovirus* genus of the *Spinareoviridae* family (3), has received significant attention due to its association with grass carp hemorrhagic disease, posing a major threat to grass carp aquaculture (30). GCRV is categorized into three genotypes, with type II being the predominant strain of GCRV circulating in China (4). Our recent study revealed that lipid droplets (LDs) are essential for GCRV-I viroplasm formation and serve as sites for GCRV-I replication and assembly (23). Moreover, we also demonstrated that GCRV-I viroplasms are formed through liquid–liquid phase separation (LLPS) of the nonstructural protein NS80, and LLPS is essential for virus replication, assembly, and immune evasion (21). However, information involved in the infection mechanisms of type II GCRV (GCRV-II) is limited. In the study, we confirmed that GCRV-II infection also induced viroplasm formation, whereas the characteristics of GCRV-II viroplasms are significantly different from those of GCRV-I and other animal reovirus, providing valuable information for the prevention and control of this virus.

Structures with liquid-like properties exhibit several typical characteristics: (i) they adopt a round shape due to surface tension (ii), they undergo fusion and fission (iii), they show rapid fluorescence recovery after photobleaching (iv), they can be dissolved by 1,6-hexanediol (1,6-HD) or propylene glycol (PG), and (v) they are not enclosed by membranes (18, 24, 31). Although GCRV-II viroplasms appear round, live-cell imaging analysis reveals that fusion and fission events are rarely observed. FRAP assays indicate that the fluorescence signal in GCRV-II viroplasms recovers more slowly than that in GCRV-I viroplasms. Moreover, GCRV-II viroplasms are resistant to 1,6-HD treatment. TEM analysis shows that GCRV-II viroplasms are clearly enclosed by membranes. Importantly, the nonstructural protein NS79, which is the key protein responsible for GCRV-II viroplasm formation, showed no characteristics of liquid-like properties. Collectively, these findings confirm that GCRV-II viroplasms are membrane-bound structures lacking liquid-like properties.

Autophagy is a highly conserved cellular process that removes long-lived proteins and damaged organelles through a lysosome-based degradation pathway (32). Autophagy often acts as a defense mechanism against viral infection (33). In our previous study, we demonstrated that GCRV-I infection induces autophagy and autophagic flux, whereas the viral-induced autophagy inhibited GCRV-I replication (34). However, it was reported that some RNA viruses subvert cellular autophagosomal machinery in order to induce autophagosome-like membrane structures for genomic RNA replication (35–37). Moreover, some plant reoviruses, such as rice gall dwarf virus (RGDV) and rice dwarf virus (RDV), hijack autophagy in insect vectors for persistent infection and viral transmission (38–40). The virion-contained membranous vesicles observed in our study are morphologically similar to autophagosomes. We therefore investigate whether GCRV-II infection induces autophagy and utilizes autophagosomes for viroplasm formation. Results revealed that GCRV-II infection induced autophagy and autophagosomes formation, as evidenced by the increased LC3B-II protein level and LC3B labeled autophagosome number. Immunofluorescence showed that NS79, VP4, or dsRNA antibodies stained viroplasms obviously colocalized with the LC3B-stained autophagosomes. Moreover, immunoelectron microscopy confirmed that the virion-contained membranous vesicles could be specifically labeled by LC3B, VP4, and dsRNA antibodies. These results suggest that GCRV-II utilizes autophagosomes for viroplasm formation and virion assembly. Nevertheless, Western blotting analysis revealed that the protein level of p62 was increased while the protein level of Lamp2 was increased after GCRV-II infection, indicating that the autophagic flux was inhibited after GCRV-II infection (41). Moreover, in cells transfected with pCMV-mCherry-GFP-LC3B, GCRV-II infection resulted in the observation of both GFP and mCherry puncta signals, which further indicates the inhibition of autophagic flux. Such inhibition may block the fusion of virion-contained autophagosomes with lysosomes to avoid virion degradation.

Outbreaks of grass carp hemorrhagic disease frequently occur in spring and summer in China, possibly due to the favorable temperatures in these seasons for viral proliferation (42). However, GCRV-II can establish subclinical persistent infection, also known as latent infection, in fish that survive the initial infection (29). Latent infection refers to a state where the host does not exhibit symptoms, yet the virus persists in specific cells without being eliminated (43). Under stress conditions, latent infection can be reactivated, producing infectious viral particles (44). Many viruses require specific conditions, such as particular host cell types and microenvironments, to establish persistent infection or latent infection (45). Therefore, investigating the detailed mechanisms of GCRV subclinical persistent infection is essential for developing strategies against GCRV infection. Consistent with previous studies, we found GCRV virions only in the brain tissues of grass carp from fish farms exposed to GCRV. Notably, these virions were enclosed within autophagosome-like vesicles. Importantly, expression levels of interferon-related genes showed no significant differences between latently infected grass carp and grass carp not exposed to GCRV. These findings suggest that GCRV-II may use autophagosomes to establish subclinical persistent infection and evade the host immune system.

The characteristics of GCRV-II infection are that it induces more than 80% mortality in yearling fish, whereas it causes no CPE in cultured cells (11). We detected specific viral bands in GCRV-II-infected cells and confirmed that the supernatant from GCRV-II-infected cells could induce typical hemorrhagic symptoms in grass carp, implying the non-lytic release of progeny virus from GCRV-II-infected cells. However, the detailed mechanism for the non-lytic release of GCRV-II is unclear. Though TEM analysis, we found the virion-contained autophagosomes at the periphery of cell membranes, fused with the cytomembrane, or outside the cells, suggesting the GCRV-II utilizes autophagosomes for non-lytic release rather than lytic release. That may be the primary reason it could not induce CPE in culture cells. Moreover, in the kidney samples from GCRV-II-infected fish, TEM also found virus-containing autophagosomes at the periphery of kidney cell membranes, outside the cells, or located at the intercellular junctional regions between the kidney cells, suggesting GCRV-II may exploit autophagosomes as the vehicles to facilitate viral spread.

**Fig 9:**
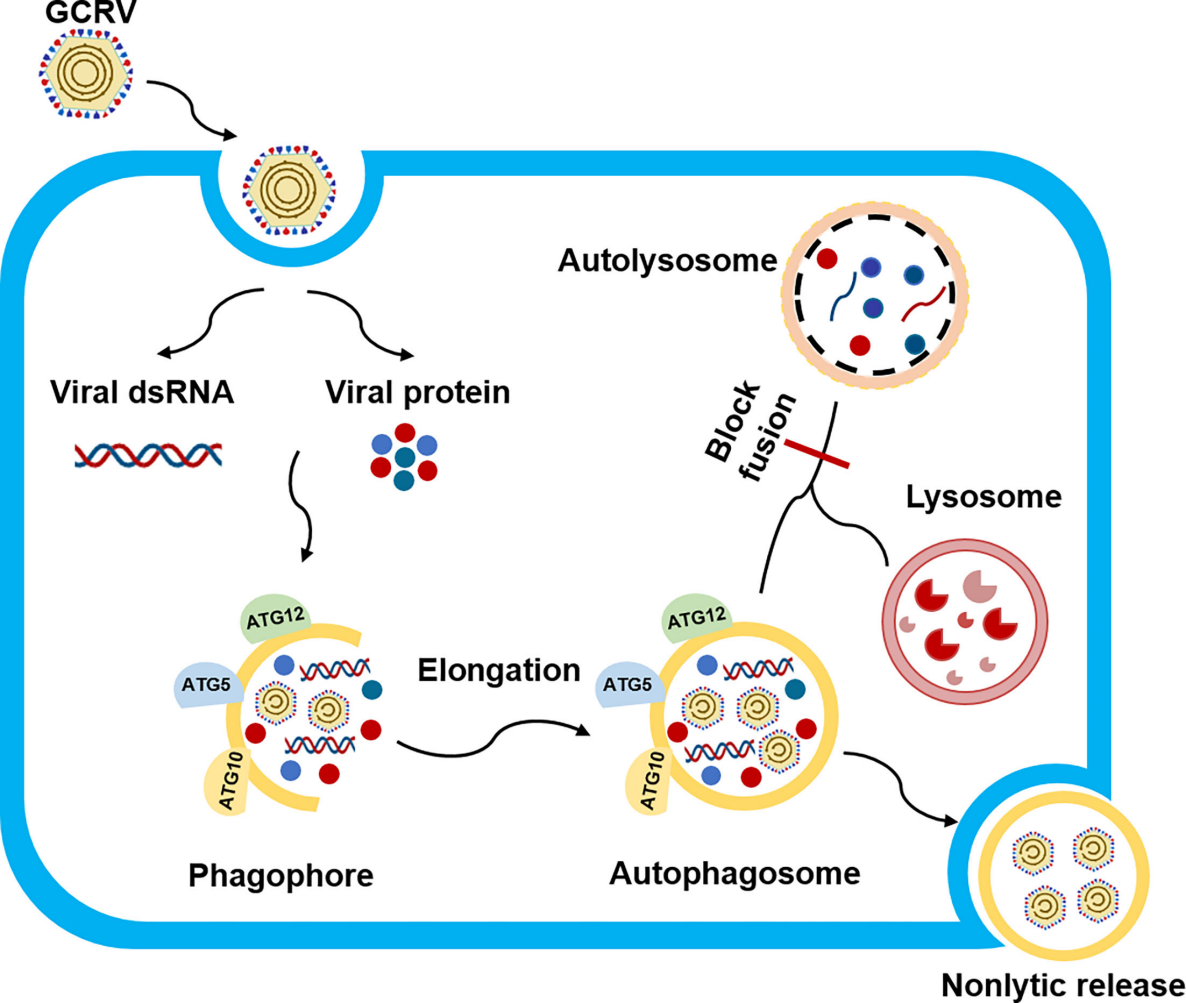
Schematic diagram of GCRV-II viroplasm formation and their role during GCRV-II infection. GCRV-II viroplasm formation and virion assembly occur in autophagosomes. GCRV-II utilizes autophagosomes for subclinical persistent infection, non-lytic release, and viral spread.

In conclusion, our study provides comprehensive insights into GCRV-II viroplasm formation and reveals their specific functions of viroplasms during GCRV-II infection. GCRV-II infection induces the formation of membranous viroplasms that lack liquid-like properties. The viroplasm formation and virion assembly occur in GCRV-II-induced autophagosomes. Moreover, GCRV-II also utilizes autophagosomes for subclinical persistent infection, non-lytic release, and viral spread (Fig. 9). Our results imply that the infection mechanism of GCRV-II is different from GCRV-I and other animal reovirus, providing valuable information for the prevention and control of this virus.

## MATERIALS AND METHODS

### Cells, fish, and viruses

Grass carp ovary (GCO) (15) and *Ctenopharyngodon idella* kidney (CIK) cells (China Center for Type Culture Collection (CCTCC), Cat# GDC0081) were cultured in M199 medium (HyClone, USA) supplemented with 10% fetal bovine serum (FBS) (NEWZERUM, New Zealand), 100 IU/mL penicillin, and 100 mg/mL streptomycin (Sigma-Aldrich, USA) under humidiﬁed conditions with 5% CO_2_ at 28°C. All cells used in this study were tested regularly for mycoplasma contamination by PCR. Healthy grass carp at the age of 3 months were used in the study, all of them from a full-sib family cultivated by Guanqiao Experimental Station, Institute of Hydrobiology, CAS. The average size is 8 ± 2 cm, and the weight is 5 ± 2 g. Before the infection experiment begins, the fish were acclimatized in aerated fresh water at 26°C–28°C for 1 week before processing. The fish were fed commercial feed (Tong Wei, China) twice daily, and the water was exchanged daily. After no abnormalities were observed, virus infection experiments were conducted. Two subtypes of grass carp reovirus (GCRV), specifically GCRV-I and GCRV-II, previously isolated and identified by our laboratory (11), were used in the study for virus infection.

### Virus infection and sample collection

Virus infection was performed in GCO cells and in grass carp. For infection in GCO cells, cells were either mock-infected or infected with GCRV-I or GCRV-II at an MOI of 1 and then harvested at different time points for live-cell imaging, immunofluorescence (IF), RT-qPCR, or TEM analysis. For infection in grass carp, about 50 grass carp were intraperitoneally injected with 200 µL of GCRV-II (2.97 × 10^3^ RNA copies/µL). Additionally, another 50 grass carp injected with the same dose of PBS served as the control group. Kidney and intestine samples were collected at different time points for immunofluorescence (IF), RT-qPCR, Western blotting, or TEM analysis.

### Antibodies and reagents

Rabbit polyclonal antibodies against grass carp IRF3 (anti-IRF3), grass carp IRF7 (anti-IRF7), GCRV-II NS79 (anti-NS79), and GCRV-II VP4 (anti-VP4) were prepared in our laboratory. Brieﬂy, the complete ORF sequences of IRF3, IRF7, NS79, and VP4 were ampliﬁed and ligated into the pET-32a expression vector. The resulting plasmids were transformed into *E. coli* BL21, and then the bacteria were induced with 1 mM IPTG for 10 hours at 20°C to express the fusion protein. The fusion proteins were puriﬁed using BeyoGold His-tag Puriﬁcation Resin (Beyotime, China), mixed with an equal volume of Freund’s adjuvant (Sigma-Aldrich, USA), and thereafter used to immunize the rabbit. The serums were collected after immunizing the rabbit three times. Mouse anti-dsRNA antibody (SCICONS, Netherlands), Alexa Fluor-594 conjugated goat anti-rabbit IgG (Cell Signaling Technology, USA), Alexa Fluor 488-conjugated goat anti-mouse IgG (Thermo Fisher Scientific, USA), and HRP-conjugated goat anti-rabbit IgG (Biosharp, China) were purchased from the indicated companies. Triton X-100, DAPI, 1,6-hexanediol (1,6-HD), rapamycin, bafilomycin A1, and 3-methyladenine were purchased from GLPBIO (GLPBIO, USA).

### Plasmids and transfection

The plasmid pEGFP-N3 was reconstructed to express the red fluorescence protein mCherry. Briefly, the open reading frame (ORF) sequence of mCherry was amplified from pmCherry-C1 (Table S1). The PCR product was digested with BamH I and Not I and then inserted into pEGFP-N3, which was treated with the same enzymes. The resulting plasmid was named pmCherry-N3, in which the EGFP ORF was replaced by mCherry.

To express the 11 GCRV-II-encoded proteins fused with EGFP or mCherry tag, we initially amplified the ORF sequences of these proteins from GCRV-II genomic dsRNA through RT-PCR. Subsequently, the PCR products were subcloned into the pEGFP-N3 or pmCherry-N3 vector employing the ClonExpress II One-Step Cloning Kit (Vazyme, China). All resulting plasmids were validated through DNA sequencing. A list of primers utilized for plasmid construction can be found in Table S1.

Transfection was performed as previously described, but with some modifications (23). GCO cells grown in glass-bottomed cell culture dishes were transfected with plasmids using the Lipofectamine 3000 transfection reagent (Thermo Fisher Scientific, USA) according to the manufacturer’s instructions. After 24 hpt, cells were ﬁxed with 4% paraformaldehyde (Beyotime, China), permeabilized with 0.2% Triton X-100, and stained with DAPI. Finally, the cells were mounted in 50% glycerol and observed under Leica TCS SP8 STED laser scanning confocal microscope (Leica, Germany) using 63 × oil objective.

### Immunofluorescence microscopy

Immunofluorescence microscopy was employed to detect the viroplasms or autophagosomes in GCRV-II-infected cells or tissues. Cells or tissues were fixed with 4% paraformaldehyde for 30 minutes. Fixed cells or tissues were permeabilized with 0.2% Triton X-100 and then blocked in 10% normal goat serum (Beyotime, China) at temperature for 1 hour. The cells were incubated with primary antibodies diluted in 1% normal goat serum for 2 hours, rinsed three times for 10 minutes each with PBS containing 1% normal goat serum, and then incubated with secondary antibodies. DAPI staining was used to visualize the nuclei. Finally, the cell tissues were rinsed with PBS, mounted with 50% glycerol, and visualized under the Leica TCS SP8 STED laser scanning confocal microscope (Leica, Germany) using 63 × oil objective. The images were further analyzed with Photoshop and ImageJ software.

### Live-cell imaging

GCO cells grown in glass-bottomed cell culture dishes were cultured in M199 medium without phenol red (Gibco, USA). Cells were transfected with the NS38-EGFP plasmid and then infected with 1 MOI of GCRV-I or GCRV-II. At 12 hpi, the infected cells were placed on a heated stage (28°C) and supplemented with warmed humidified air containing 5% CO2. Cell samples were observed under the Leica TCS SP8 STED laser scanning confocal microscope (Leica, Germany) using 63 × oil objective, and images and movies were recorded at indicated time points by Leica LAS AF lite software.

### Fluorescence recovery after photobleaching assay

Fluorescence recovery after photobleaching (FRAP) assay was performed using the FRAP module of the Leica TCS SP8 STED laser scanning confocal microscope with a 63 × oil objective. Cells were transfected with the NS38-EGFP plasmid and then infected with 1 MOI of GCRV-I or GCRV-II. At 12 hpi, globular inclusions of NS38-EGFP were bleached using a 473 nm laser beam. Regions of interest (ROIs) were photobleached to approximately 10%–20% of their original intensity, and then ﬂuorescence recovery was determined over time. Time-lapse images were collected every 15 seconds and in a total of 300 seconds. The fluorescence intensity of the photobleached areas was normalized to the same areas before photobleaching. GraphPad Prism software was used to plot and analyze the FRAP results.

### RT-qPCR

RT-qPCR was used to investigate the mRNA expression levels of interferon-related genes (IRF3, IRF7, IFN1, and IFN3). Total RNA was isolated using the AG RNAex Pro Reagent (Accurate Biology, China), and first-strand cDNA was obtained using a HiScript III 1st Strand cDNA Synthesis Kit (Vazyme, China). RT-qPCR was performed using a fluorescence quantitative PCR instrument (Bio-Rad, USA). Each reaction mixture contained 0.8 µL forward and reverse primers (for each primer), 1 µL cDNA template, 10 µL 2 × ChamQ SYBR qPCR Master Mix (Vazyme, China), and 7.4 µL ddH2O. Three replicates were performed for each sample, and β-actin was used as an internal control for the normalization of gene expression. The program was as follows: 95°C for 10 seconds; 40 cycles of 95°C for 15 seconds, 56°C for 30 seconds, and 72°C for 30 seconds; and melt curve construction. The relative expression levels were calculated using the 2^-∆∆Ct^ method (46). Data are presented as mean (*n* ≥ 3) ±standard deviation (SD) of three replicates.

### Western blotting

Western blotting was employed to examine the protein expression levels of interferon regulatory factors (IRF3 and IRF7) in grass carp. Cells or tissues were lysed in RIPA lysis buﬀer (Beyotime, China) on ice for 30 minutes and collected by centrifuge at 12,000 × *g* at 4°C for 10 minutes. Proteins were separated by 15% SDS-PAGE and transferred to a PVDF membrane (Millipore, USA). The membranes were washed with Tris-Buﬀered Saline Tween-20 (TBST) buffer (Solarbio, China) and then incubated with 5% nonfat milk powder, followed by incubation with primary antibodies at a 1:1000 dilution overnight. After washing in TBST, PVDF membranes were incubated with HRP-conjugated goat anti-rabbit IgG (Biosharp, China) at room temperature for 1 hour. Finally, the immunoblot signals were detected using an HRP-DAB Chromogenic Kit (Tiangen, China).

### Transmission electron microscopy

Transmission electron microscopy (TEM) experiments were performed as previously described, with some modifications (23). GCO cells or grass carp were either mock-infected or infected with GCRV-II. Cells or kidney samples were harvested at 24 or 48 hpi by centrifugation at 2000 × *g* for 5 minutes. The pellets were pre-fixed with 2.5% glutaraldehyde for 24 hours at 4°C, then followed by post-fixation with 1% osmium tetroxide (OsO_4_) for 2 hours at 4°C. The samples were dehydrated stepwise in a graded series of ethanol and embedded in the epoxy resin Epon-812 overnight. The specimens were cut using a Leica DMIRB ultrathin microtome at 70 nm thickness, double stained with uranyl acetate and lead citrate, and observed with an HC-1 80.0 KV Hitachi TEM system (Hitachi, Japan).

### Immunoelectron microscopy

The kidney samples from GCRV-II-infected or control fish were fixed, dehydrated, and embedded, and the ultrathin sections were cut as previously in the above. Then, the sections were immunolabeled with antibodies against LC3B, VP4, or dsRNA as the primary antibodies, followed by treatment with goat anti-rabbit IgG or goat anti-mouse IgG conjugated with 10-nm-diameter gold particles as the secondary antibodies (Abcam, UK). Thin sections were examined with an HC-1 80.0 KV Hitachi TEM system (Hitachi, Japan).

### Hematoxylin and eosin staining

Hematoxylin and eosin staining was done as described previously (47). Brieﬂy, liver and intestine samples of grass carp from different groups were ﬁxed in Bouin’s fixative overnight at 4°C. Following dehydration, the samples were embedded in HistoResin (Leica). Serial sections of 4 mm thickness were cut using a microtome (Leica), dried on slides at 42°C overnight, stained with hematoxylin and eosin (H&E), mounted in Permount (Fisher), and imaged with phase contrast with a 63 × oil immersion objective lens.

### Statistical analysis

All experiments were performed at least three times. One-way analysis of variance (ANOVA) and unpaired two-tailed Student’s *t*-test were used to analyze statistical significance. Data are represented as mean (*n* ≥ 3) ±standard deviation (SD). Statistical significance is depicted with stars (* indicates *P* < 0.05, ** indicates *P* < 0.01, ns indicates no significant difference).

## Data Availability

The data generated in the study are available in the published article and its online supplemental material.

## SUPPLEMENTAL MATERIAL

Supplemental material jvi.00352-25-s0001.pdf Figures S1 to S4; Table S1.

ASM does not own the copyrights to Supplemental Material that may be linked to, or accessed through, an article. The authors have granted ASM a non-exclusive, world-wide license to publish the Supplemental Material files. Please contact the corresponding author directly for reuse.
