# Supplementary material for: Type II grass carp reovirus utilizes autophagosomes for viroplasm formation and subclinical persistent infection
Source: J Virol. 2025 Apr 2;99(5):e00352-25. doi: 10.1128/jvi.00352-25 (PMC12090803; doi:10.1128/jvi.00352-25)
Supplement: Supplemental material — Figures S1 to S4; Table S1. [file jvi.00352-25-s0001.pdf]

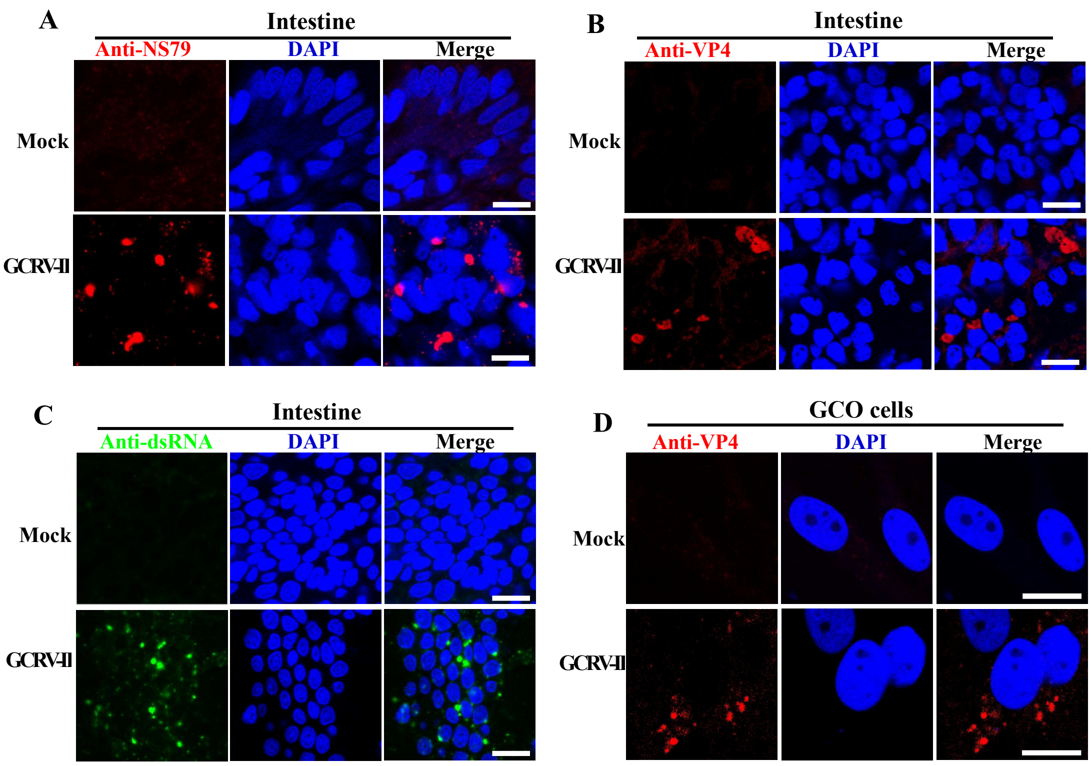


**Figure S1. GCRV-II infection induces the formation of viroplasms. (A-C)** Immunofluorescence analysis of intestine samples from GCRV-II infected fish by using antibodies against N79 (A), VP4 (B), or dsRNA (C), respectively. Grass carp were mock infected or infected with GCRV-II and intestine samples were collected for immunofluorescence analysis. Scale bar=10 μm. **(D)** Immunofluorescence analysis of GCRV-II GCO cells using antibodies against VP4. GCO cells were mock infected or infected with GCRV-II and then stained with anti-VP4 antibody. Scale bar=10 μm.

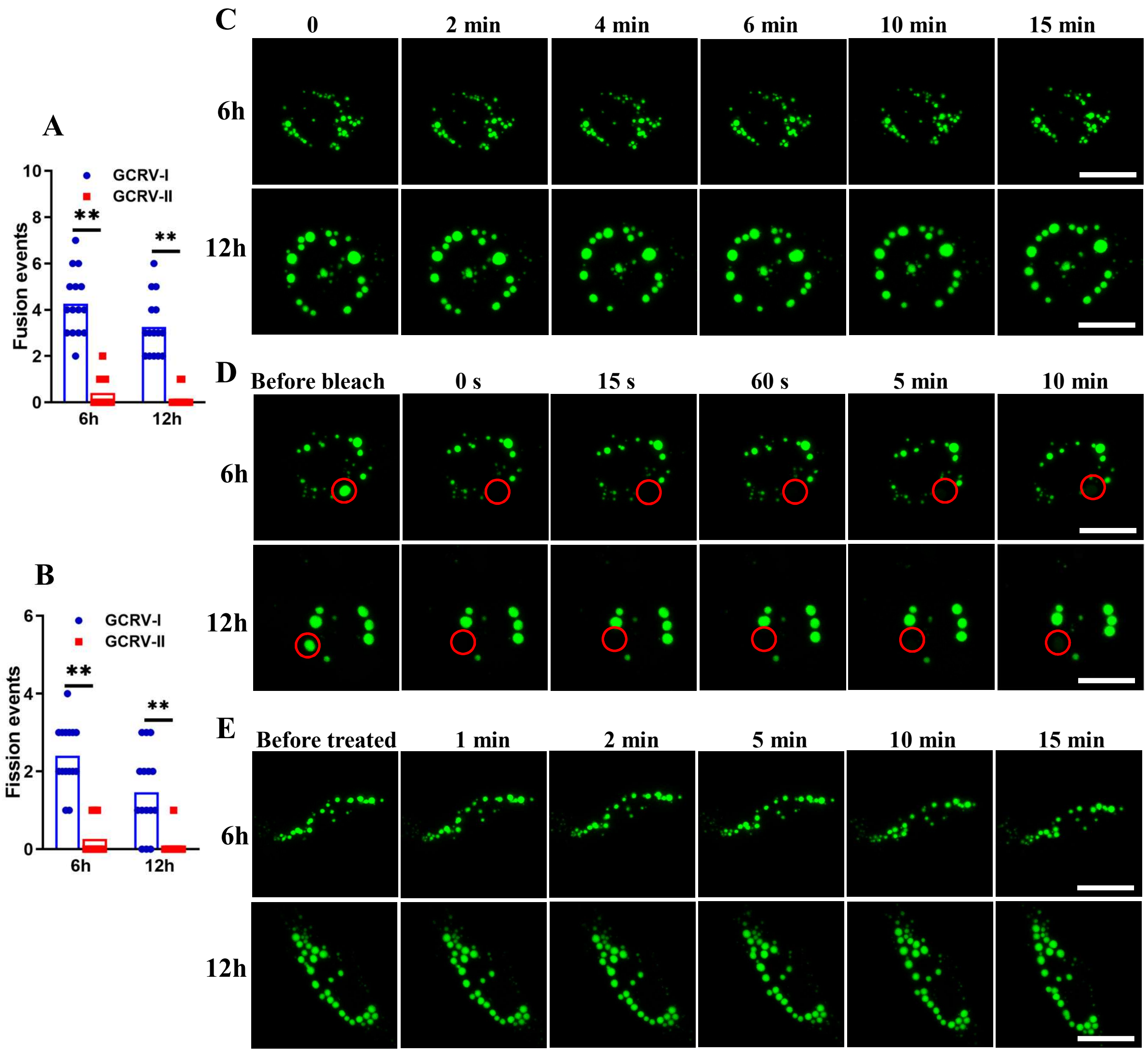


**Figure S2. GCRV-II viroplasms lack liquid-like properties. (A, B)** Quantitative analysis the number of fusion (A) and fission (B) events in GCRV-I or GCRV-II viroplasms at 6 and 12 hours post infection. Data are represented as mean (n=15) ± SD. ** indicates $P < 0.01$. **(C)** Live-cell imaging analysis of NS79-EGFP formed VLSs at 6 or 12 hours post transfection (hpt). GCO cells were transfected with NS79-EGFP and collected at 6 or 12 hpt for live-cell imaging analysis. Scale bar=10 μm. **(D)** FRAP assay of NS79-EGFP formed VLSs at 6 or 12 hpt. GCO cells were transfected with NS79-EGFP plasmid and collected at 6 or 12 hpt for FRAP assay. Scale bar=10 μm. **(E)** Investigate the liquid-like properties of NS79-EGFP formed VLSs by 1,6-HD treatment. GCO cells were transfected with NS79-EGFP plasmid and then treated with 4% 1,6-HD at 6 or 12 hpt. Scale bar=10 μm.

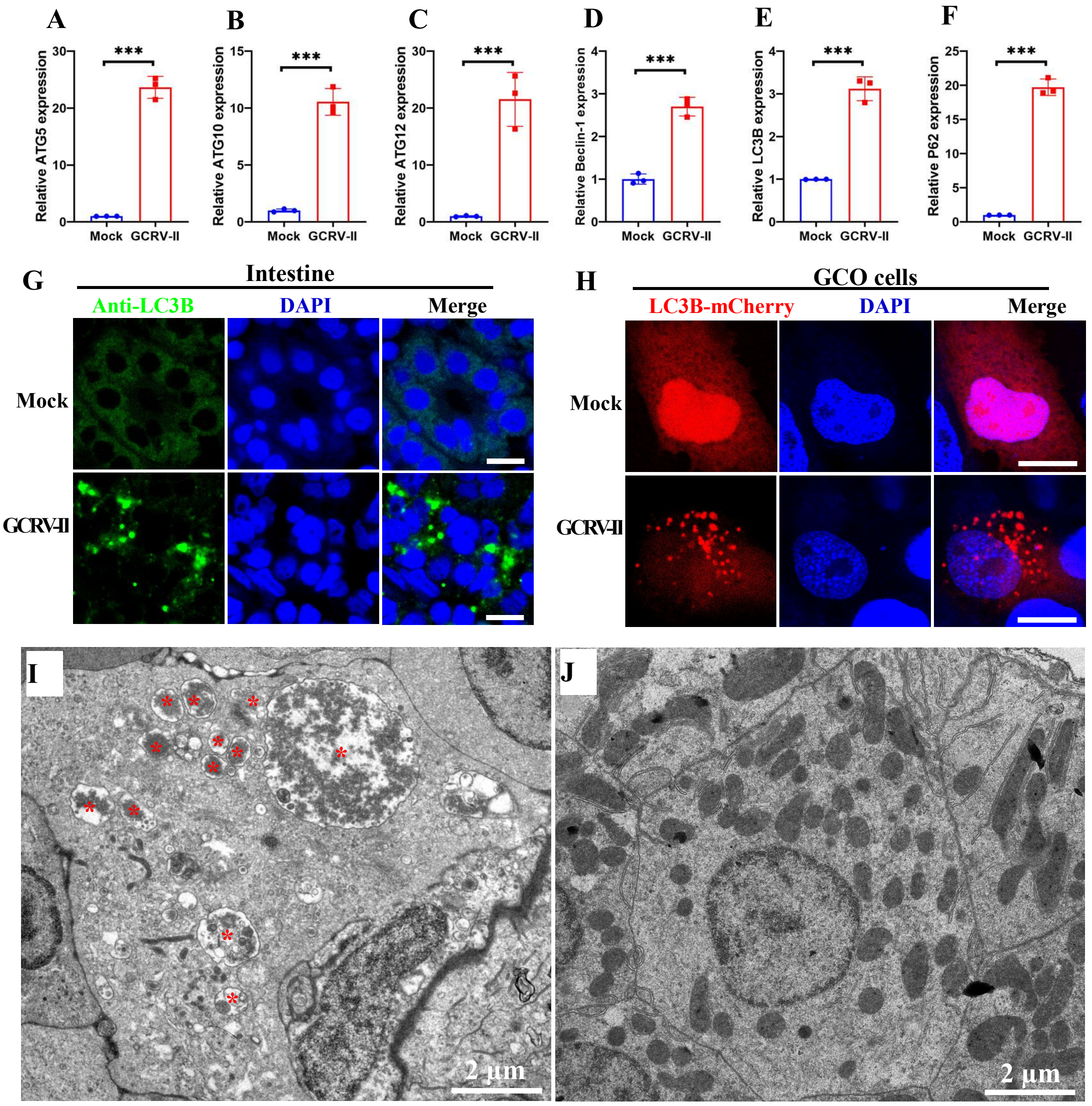


**Figure S3. GCRV-II infection induces autophagy and autophagosome formation.** **(A-F)** RT-qPCR analysis the expression levels of autophagy-related genes (ATG5, ATG10, ATG12, LC3B, Beclin-1, and P62) in kidney samples from GCRV-II infected or control fish. Data are represented as mean (n=3) ± SD. ** indicates $P < 0.01$. **(G)** Immunofluorescence analysis of intestine samples from GCRV-II infected or control fish by using antibodies against LC3B. Scale bar=10 µm. (**H**) The localization patterns of LC3B-mCherry in the absence or presence of GCRV-II infection. GCO cells were transfected with LC3B-mCherry plasmid and then mock infected or infected with GCRV-II and harvested for fluorescence observation. Scale bar = 10 µm. **(I, J)** TEM analysis kidney samples from GCRV-II infected or control fish. The asterisks indicate autophagosome-like vesicles in GCRV infected kidney samples. Scale bar = 2 µm.

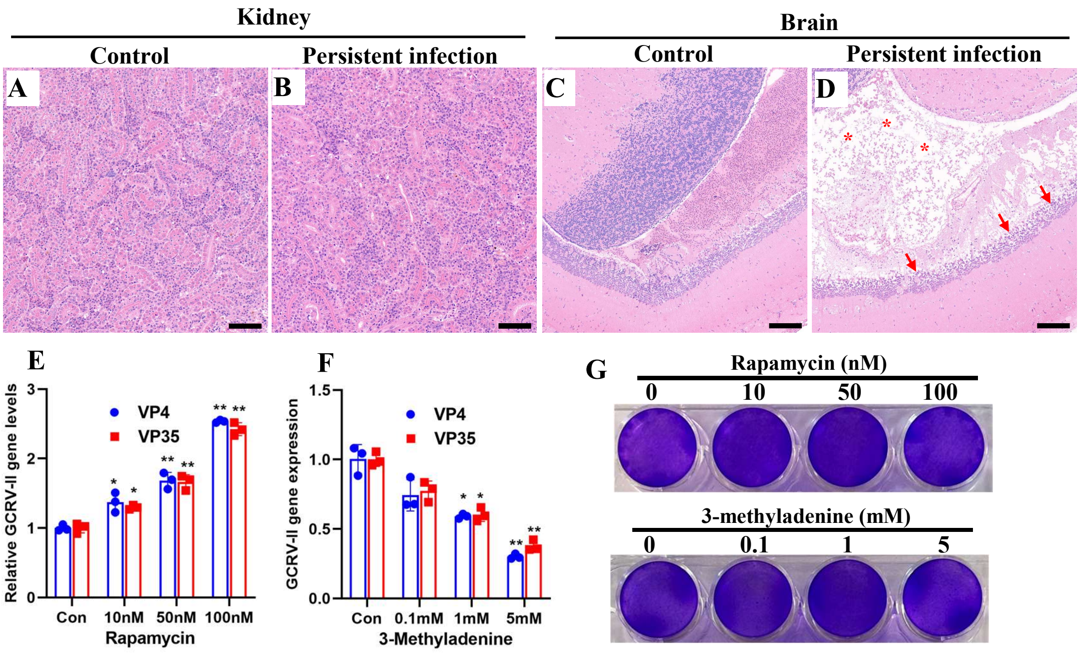


**Figure S4. GCRV-II utilizes autophagosomes for subclinical persistent infection and nonlytic release. (A-D)** Histological section analysis the kidney and brain tissues from GCRV-II subclinical persistent infected fish and control fish. Scale bars = 50 (A and B) and 100 μm (C and D). The asterisks indicate the loose brain matrix, while the red arrows indicate the cell necrosis. (**E, F**) RT-qPCR detection the relative copy number of GCRV-II in supernatants from rapamycin (E) or 3-Methyladenine (F) treated cells. Data are represented as mean (n=3) ± SD. * indicates $P < 0.05$, ** indicates $P < 0.01$. (**G**) Analysis the cytopathic effects (CPE) in GCRV-II infected cells after treated with rapamycin or 3-methyladenine.

## Table S1. Primer sequences used in the study

| primers | Sequences (5' to 3') | usage |
|---|---|---|
| mCherry-F | CCGGGATCCATCGCCACCATGGTGAGCAAGGG | Mcherry amplification |
| mCherry-R | AGTCGCGGCCGCTCTACTTGTACAGCTC | |
| VP1-F | TCGAGCTCAAGCTTCGAATTCTGCCACCATGGCATTGTTTGGATTGAG ACTAT | VP1 amplification |
| VP1-R | CACCATGGTGGCGATGGATCCTGAGACCACACGGACTAACTCATACG | |
| VP2-F | TCGAGCTCAAGCTTCGAATTCTGCCACCATGGACCATGTGTACCAAGG CC | VP2 amplification |
| VP2-R | CACCATGGTGGCGATGGATCCCACTTTTCGCATCCACATCTCAAAG | |
| VP3-F | TCGAGCTCAAGCTTCGAATTCTGCCACCATGCATCGTCATAACAGAAC ACGTG | VP3 amplification |
| VP3-R | CACCATGGTGGCGATGGATCCCTCTACTCCCGCCATAGTTCCC | |
| NS79-F | TCGAGCTCAAGCTTCGAATTCTGCCACCATGGCGATGCGTCCGTCGTC | NS79 amplification |
| NS79-R | CACCATGGTGGCGATGGATCCATGGCCTGAGTCGTAGAACAAATC | |
| VP5-F | TCGAGCTCAAGCTTCGAATTCTGCCACCATGTTACTCATTCTGCCCAC GTACA | VP5 amplification |
| VP5-R | CACCATGGTGGCGATGGATCCATCCCCATGTGGAAGTGGTACCTC | |
| VP4-F | TCGAGCTCAAGCTTCGAATTCTGCCACCATGGGAAACGTCCAGACGA ACAG | VP4 amplification |
| VP4-R | CACCATGGTGGCGATGGATCCAGACGGAGGAGGCCAGTATCGC | |
| VP56-F | TCGAGCTCAAGCTTCGAATTCTGCCACCATGGCCACTCGTGACAGCCG | VP56 amplification |
| VP56-R | CACCATGGTGGCGATGGATCCCTTACAGCAAACTACCGTCCAATGC | |
| VP41-F | TCGAGCTCAAGCTTCGAATTCTGCCACCATGTATCTGGAACTGTTCAT CGCTG | VP41 amplification |
| VP41-R | CACCATGGTGGCGATGGATCCCGGGCTCTTAGCCTTTGCCTTG | |
| VP6-F | TCGAGCTCAAGCTTCGAATTCTGCCACCATGGAGCGATCCACTTACAA TATCT | VP6 amplification |
| VP6-R | CACCATGGTGGCGATGGATCCAGGGAATAAGCGATCCAACACC | |
| NS38-F | TCGAGCTCAAGCTTCGAATTCTGCCACCATGGCGGGTGTGTCTCTCAA C | NS38 amplification |
| NS38-R | CACCATGGTGGCGATGGATCCCAGCATCTGCGCAAATATACGTC | |
| VP35-F | TCGAGCTCAAGCTTCGAATTCTGCCACCATGGAATCAGCAAAACCATT GAC | VP35 amplification |
| VP35-R | CACCATGGTGGCGATGGATCCCTGTCCCTGGATCTCAGGTTTGA | |
| IRF3-F | GCCATGGCTGATATCGGATCCATGACCCATCCAAAACCGCTC | IRF3 amplification |
| IRF3-R | CTCGAGTGCGGCCGCAAGCTTTCACTTGGTGTCACACAACTC | |
| IRF7-F | GCCATGGCTGATATCGGATCCATGGCAGCGATGCAGAGCAG | IRF7 amplification |
| IRF7-R | CTCGAGTGCGGCCGCAAGCTTTTAGTCCATTGAAGGCAGACCC | |
| GCRV-II-F | AGCGCAGCAGGCAATTACTATCT | RT-PCR of actin |
| GCRV-II-R | ATCTGCTGGTAATGCGGAACG | |
| actin-F | AGCCATCCTTCTTGGGTATG | RT-PCR of actin |
| actin-R | GGTGGGGCGATGATCTTGAT | |
| qIRF3-F | AAAATGTGGACACTGACGGACC | QPCR of IRF3 |
| qIRF3-R | CCAACACCATCTTCCCTTTGTAATA | |
| qIRF7-F | AATACGCTTTCCAACCACCG | QPCR of IRF7 |
| qIRF7-R | CAGTTTTCTGGGCTCTGGGTT | |

| | | |
|---|---|---|
| qIFN1-F | AAGCAACGAGTCTTTGAGCCT | QPCR of IFN1 |
| qIFN1-R | GCGTCCTGGAAATGACACCT | |
| qIFN3-F | TACATTTATAGAGACTGCGGGTGG | QPCR of IFN3 |
| qIFN3-R | TGGAGTGTCTGGTAAACAGCCTT | |
| qVP4-F | AGCATCAGCAATGCAGGAGT | QPCR of *VP4* |
| qVP4-R | CGGGACAACATCCAAGACGA | |
| qVP35-F | GGTGACTGTATCCAACGGCT | QPCR of *VP35* |
| qVP35-R | AATGTGAGTAACCGCAGCGA | |
| qβ-actin-F | AGCCATCCTTCTTGGGTATG | QPCR of *β-actin* |
| qβ-actin-R | GGTGGGGCGATGATCTTGAT | |
